# Deciphering shared molecular dysregulation across Parkinson’s disease variants using a multi-modal network-based data integration and analysis

**DOI:** 10.1038/s41531-025-00914-3

**Published:** 2025-03-31

**Authors:** Alise Zagare, Irina Balaur, Adrien Rougny, Claudia Saraiva, Matthieu Gobin, Anna S. Monzel, Soumyabrata Ghosh, Venkata P. Satagopam, Jens C. Schwamborn

**Affiliations:** Luxembourg Centre for Systems Biomedicine (LCSB), University of Luxembourg, Esch-sur-Alzette, Luxembourg

**Keywords:** Cell biology, Computational biology and bioinformatics, Neuroscience, Systems biology

## Abstract

Parkinson’s disease (PD) is a progressive neurodegenerative disorder with no effective treatment. Advances in neuroscience and systems biomedicine now enable the use of complex patient-specific in vitro disease models and cutting-edge computational tools for data integration, enhancing our understanding of complex PD mechanisms. To explore common biomedical features across monogenic PD forms, we developed a knowledge graph (KG) by integrating previously published high-content imaging and RNA sequencing data of PD patient-specific midbrain organoids harbouring LRRK2-G2019S, SNCA triplication, GBA-N370S or MIRO1-R272Q mutations with publicly available biological data. Furthermore, we generated a single-cell RNA sequencing dataset of midbrain organoids derived from idiopathic PD patients (IPD) to stratify IPD patients within the spectrum of monogenic forms of PD. Despite the high degree of PD heterogeneity, we found that common transcriptomic dysregulation in monogenic PD forms is reflected in glial cells of IPD patient midbrain organoids. In addition, dysregulation in ROBO signalling might be involved in shared pathophysiology between monogenic PD and IPD cases.

## Introduction

The characteristic motor impairment in Parkinson’s disease (PD) is attributed to the gradual degeneration of dopaminergic neurons in the midbrain, yet the exact cause of this neuronal loss remains unknown^1^. Furthermore, monogenic cases account for only approximately 10% of PD, leaving the majority of cases classified as idiopathic (IPD)^2,3^. Identification of shared dysregulated molecular pathways between monogenic and IPD cases holds significant importance in understanding disease mechanisms enabling the development of therapeutic strategies that could be applicable across multiple PD patient groups.

In recent years, there has been a significant advancement in high-throughput experimental technologies, allowing scientists to generate large amounts of biomedical data to investigate complex disease mechanisms^4^. However, this data is often collected and provided in various formats across different studies, hindering data integration and secondary analyses^4,5^. Therefore, in systems biomedicine, harmonisation and standardisation of research outputs are crucial to maximise the interpretability and reproducibility of results, and to facilitate comprehensive data integration across various research studies and experiments. Graph databases (GDBs) have been used for this task in systems biomedicine due to their flexibility (i) to represent naturally the biomedical information and to integrate large sets of heterogeneous data types (including omics, clinical, imaging, sensor data, etc.), (ii) to capture complex data inter-relationships and (iii) to provide support for network-based analysis and modelling of biomedical data^6–9^. This approach is particularly appealing in studying complex diseases, such as PD, where cause-effect relationships remain difficult to decipher^10^. In particular, the integration of large amounts of heterogeneous data in knowledge graphs (KGs) enables the discovery of new relationships using reasoning frameworks^11^ such as machine learning (ML)^12^, with various applications in biomedicine^13^. For example, KGs have been developed for network-based analysis of disease-specific data^14,15^, exploration of disease mechanisms, comorbidities, and risk factors^16^, and development and repurposing of drugs^17^. In the context of PD, several KGs have been developed for the identification of novel mechanisms and drug targets, either with a large biomedical scope only applied to PD^18–23^, or focusing on data related to neurodegenerative diseases in general^14,24,25^.

In this study, we developed the PD-KG, a PD-related data-centric knowledge graph. The PD-KG was built by integrating existing high-content imaging and RNA sequencing PD data with biological data from major public resources (e.g. Reactome^26^, IntAct^27^, DisGeNet^28^, DGIdb^29^, UniProtKB^30^) that contextualize it. The integrated experimental data was previously acquired from midbrain organoids generated from induced pluripotent stem cells (iPSCs) of PD patients harbouring LRRK2-G2019S^31,32^, SNCA triplication^33^, GBA-N370S^34^ or MIRO1-R272Q^35^ mutations. We further performed a network-based analysis on the PD-KG, focusing on the identification of common dysregulated molecular features across multiple PD-associated mutations. This analysis resulted in a comprehensive overview of the pathways, gene interaction partners and drugs shared between the datasets, revealing 25 genes with shared dysregulation in at least two monogenic PD cases. Notably, these 25 genes also demonstrated differential expression in the glial cells (radial glia, astrocytes and oligodendrocytes) of IPD patients, as observed in a newly generated single-cell RNA sequencing experiment. Additionally, our analysis suggests that dysregulation in ROBO signalling and altered axonogenesis may represent a potential shared disease mechanism between IPD and monogenic PD. Importantly, our work also provides a harmonised and integrated multimodal dataset comprising transcriptomics and high-content imaging data from monogenic PD and IPD-specific midbrain organoids, ready to use for future studies.

## Results

### PD knowledge graph (PD-KG)

**Fig. 1:**
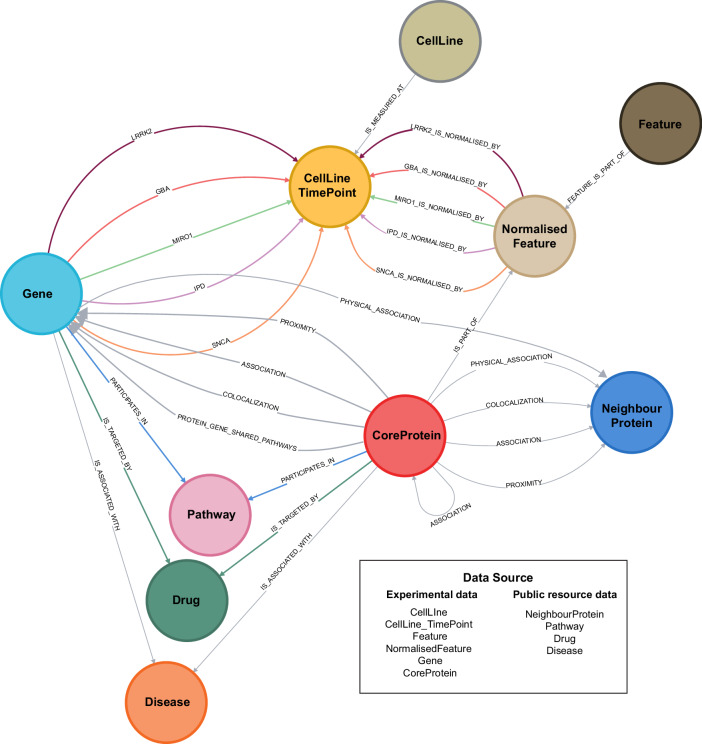
The graph data model for the PD-KG. The data types given by biological concepts such as proteins, genes, pathways, diseases, drugs, and cell lines are represented as nodes and their inter-relationships (e.g. gene-pathway involvement) as edges in the underlying graph.

**Fig. 2:**
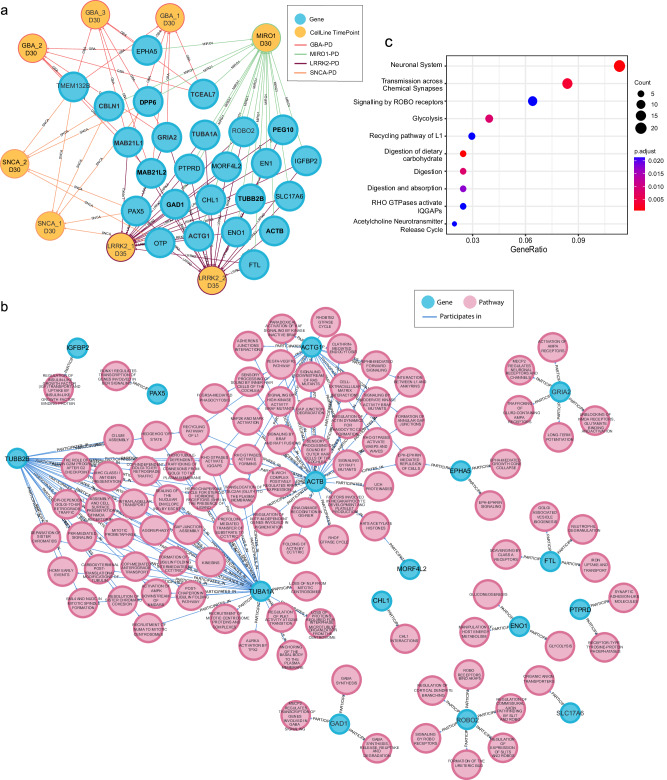
In silico analysis reveals shared genes and pathways between monogenic PD datasets. **a** Visual representation of PD-KG subset demonstrating relationships between cell lines used for midbrain organoid generation in individual experiments (yellow nodes) and genes from the top 100 significantly differentially expressed lists (blue nodes). D30 and D35 indicate that transcriptomics analysis was done for midbrain organoids at day 30 or 35 of culture, respectively. The colour of the edges indicates different datasets (GBA-red, LRRK2-purple, MIRO1-green, and SNCA-orange). **b** Visual representation of PD-KG subset demonstrating shared genes between the four PD datasets involvement in pathways (pathway source: Reactome). Blue nodes—genes; pink nodes—pathways. **c** Pathway overrepresentation analysis of the merged list of the top 100 significantly differentially expressed genes from all four PD datasets.

We developed the PD-KG, integrating the high-content imaging data and the top 100 significantly differentially expressed genes (DEGs) from RNA sequencing experiments available in the previously published experimental datasets on PD patient-specific midbrain organoids^31,33–35^ with biological data from public resources (e.g. Reactome^26^, IntAct^27^, DisGeNet^28^, Drug–Gene Interaction Database (DGIdb)^29^, UniProtKB^30^) corresponding to multiple biomedical layers (e.g. disease associations, drug targets, pathway involvements, protein-protein interactions). The available transcriptomics data were obtained from a single time point of organoid culture, whereas the imaging datasets were collected at various organoid culture time points, ranging from day 15 to day 180. Concepts (such as genes/proteins, pathways, and drugs) were represented as nodes and relationships among concepts (e.g. protein-disease association, protein-drug target) as edges in the underlying graph. Its data model is shown in Fig. 1. For example, the transcriptomics measurement of the TCEAL7 gene in the GBA_3 cell line at the Day 30 time point in the GBA-PD dataset is shown by the “GBA” edge (relationship) connecting the TCEAL7 gene and the “GBA_3 D30” CellLineTimePoint nodes (Fig. 2a). Annotations (such as the cell line provenience for imaging data, and the mapping between gene symbols and unique UniProt identifiers to enhance interoperability), were captured as attributes (properties) of nodes and edges in the graph. The PD-KG contains 3610 nodes and 8512 edges (relationships). Details on the semantics of the nodes and relationship types together with a brief summary of the connections between genes (from both core proteins and transcriptomics sets) with other biological entries, (such as pathways, drugs, proteins etc.), respectively, are provided in Supplementary Tables 1–3. A comparison of the PD-KG with other KGs for neurodegenerative diseases is also available in Supplementary Table 4. Among these KGs, the PD-KG is the only data-centric KG specifically targeting the contextualisation of PD molecular features being developed by the integration of PD-related experimental data with knowledge from other well-established public biological data resources.

### In silico analysis reveals shared genes and molecular pathways between monogenic PD cases

Integration of data from previously published studies on PD patient-specific midbrain organoids allowed us to explore the similarities in gene and protein expression patterns between the four different PD forms caused by a pathogenic variant in the LRRK2, SNCA, GBA or RHOT1 (encoding MIRO1) genes. We focused on the top 100 significant DEGs from each experimental dataset, which we believe adequately represent the key transcriptomic signatures underlying PD-associated phenotypes, and we aggregated a combined set of 400 DEGs across all four studies. First, we noticed that there is no single shared DEG between all four monogenic PD cases. However, we observed 25 genes shared between at least two of the datasets (Fig. 2a and Supplementary Table 5). Interestingly, 15 of these 25 genes were shared between LRRK2-PD and MIRO1-PD datasets, suggesting a higher similarity of transcriptomic dysregulation between these two monogenic PD forms. Furthermore, only 12 out of the total 25 shared genes showed the same expression direction (UP or DOWN) across the compared groups (Fig. 2a—highlighted in bold and Supplementary Table 6). The set of 13 genes with different regulation directions suggests that similar molecular processes may be differentially regulated in different PD cases.

Integration with public data repositories allowed us to further explore the experimental data. We used the PD-KG to identify common pathways between the 25 genes of interest shared between at least two datasets. Sixteen genes were reported as involved in at least one Reactome pathway (Fig. 2b). Genes MORF4L2, EPHA5, ACTB, ACTG1, TUBA1A and TUBB2B showed the highest involvement rate in a variety of cellular pathways demonstrating direct and indirect connections forming a larger network of relationships. In total these six genes through the gene-pathway interactions were associated with 69 pathways, including the “Recycling Pathway of L1”, “Translocation of SLC24 (GLUT4) to the plasma membrane”, “RHO GTPases activate IQGAPS”, “RHO GTPases activate FORMINS”, and “EPH-EPHRIN mediated repulsion of cells” (Supplementary Table 7).

Further, we conducted an over-representation analysis (ORA)^36^, considering the combined list of the top 100 most significant DEGs from individual experiments. While the PD-KG indicated specifically the Reactome pathways involving the 25 shared DEGs, the ORA analysis explored whether both the shared and unique DEGs from each study are involved in similar pathways. The ten most enriched pathways were associated with the neuronal system, synaptic function, ROBO signalling, metabolism (glycolysis, digestion of carbohydrates, digestion and absorption), IQ motif-containing GTPase-activating proteins (IQGAPs) and acetylcholine release cycle (Fig. 2c). The overlap between several pathways associated with shared and experiment-specific transcriptomic features suggests commonalities in pathway-level dysregulation among the four PD-associated mutations.

The available high-content imaging data from the four published studies were inconsistent due to the custom selection of proteins for imaging analysis and the difference in the relevant time points considered in each individual experiment. Usually, proteins for immunostaining analysis are selected based on previous knowledge of predicted mutation-associated phenotypes^31,33–35^. Across all four independent experiments, a set of 12 core proteins was analysed, from which all were integrated into the PD-KG (Supplementary Table 8). The tyrosine hydroxylase (TH, UNIPROT id: P07101) was the only core protein common to all datasets. TH is a rate-limiting enzyme in dopamine synthesis and, thus, an essential marker of dopaminergic neurons, which is the main neuronal population affected in PD. We compared how levels of dopaminergic neurons and their fragmentation, as an early sign of neurodegeneration, change over time across all datasets. This allowed us to confirm that all PD patient midbrain organoids display reduced levels of TH dopaminergic neurons, consistent with findings from the original studies. Additionally, these organoids showed increased fragmentation compared to healthy control midbrain organoid samples up to day 120 of organoid culture (Supplementary Figs. 1 and 2).

Given the limitations that hinder other core protein abundance comparison across all four datasets, we used the PD-KG to explore the pathways involving both the core proteins from imaging data and the top 100 significant DEGs within the same experiment, revealing genotype-phenotype relationships and the relevance of the analysed proteins (Supplementary Fig. 3a–c and Supplementary Table 9). We observed that in the LRRK2-PD experiment, no connections between the top DEGs and the core proteins were detectable. The other three datasets shared several core proteins, namely TUBB3, TH, GFAP, and S100b, and we focused on their pathway involvement, as follows. In the MIRO1 and SNCA datasets, the GFAP and S100b glial markers from imaging data shared pathways with the cytoskeleton filament-associated STMN1 and GFAP genes, suggesting that glial cell development or maturity might be affected in these forms of PD (Supplementary Fig. 3a, b). In the GBA study, relationships were identified between the TH and SOX2 proteins and one or several significant DEGs, respectively. This indicates that dysregulation in stem cells and dopaminergic neurons plays a crucial role, particularly in the development of GBA-PD as also reported in the original study^34^ (Supplementary Fig. 3c). In all three datasets, the TUBB3 protein shared pathways with several significant DEGs, including CDKN1A, ACTB, VASH2, L1CAM, CTSF, PMSD5, and others (Supplementary Fig. 3a–c). TUBB3 is a neuronal protein that forms microtubules and is involved in neurogenesis and axon guidance. Overall, associations between glial proteins and neuronal TUBB3 with the significant DEGs involved in cytoskeleton dynamics are consistent with ORA results, suggesting common PD dysregulation in ROBO signalling, also involved in axon guidance and cytoskeleton organisation^37^. Although, in the LRRK2 dataset we did not find shared pathways between analysed core proteins and the top significant DEGs several isoforms of tubulins, such as TUBA1A, TUBB2B and TUBA1B were found in the list of top 100 significant DEGs, further suggesting that disrupted microtubule cytoskeleton organisation and altered axon guidance might be shared mechanisms between the four monogenic PD cases.

### Idiopathic PD shares transcriptomic dysregulation with monogenic PD cases

The integration of transcriptomic data of four different PD-associated mutations in the PD-KG revealed 25 significantly dysregulated genes shared between at least two of the mutations. Moreover, we were able to identify dysregulation in tubulins and ROBO signalling indicating cytoskeleton organisation and axonal guidance as potential common PD mechanisms across different PD-associated mutations. This suggests that despite the overwhelming PD heterogeneity, there are some similarities in the transcriptomic landscape between LRRK2-G2019S, 3xSNCA, GBA-N370S and MIRO1-R272Q monogenic PD forms. We further investigated if there are also similarities between these four monogenic PD cases and IPD.

**Fig. 3:**
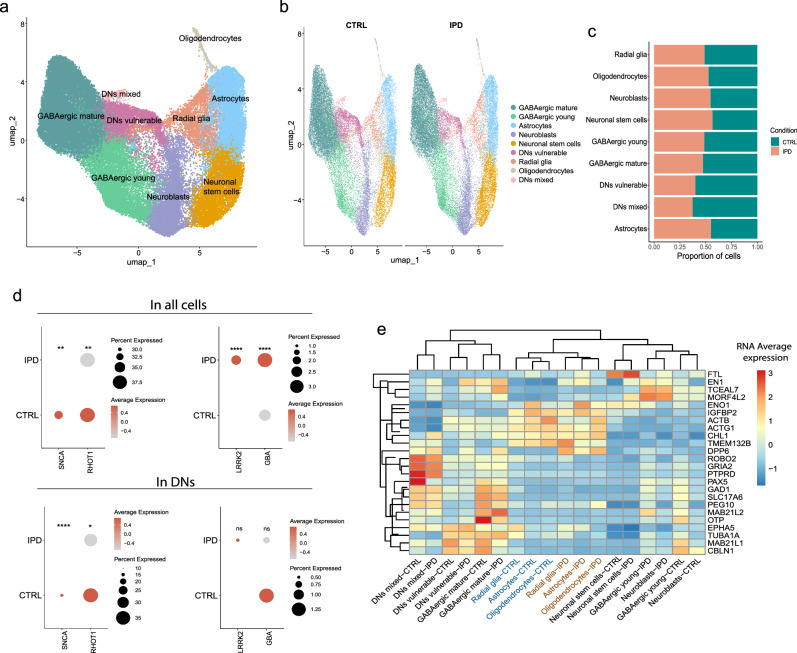
Single-cell RNA sequencing analysis of IPD-patient-specific midbrain organoids. **a** UMAP representation of identified cellular populations. Every dot represents a single cell and colours indicate different cellular populations. **b** UMAP representations of cellular populations separated by healthy control (CTRL) and IPD samples. Every dot represents a single cell and colours indicate different cellular populations. **c** Proportion of each cell population in CTRL and IPD conditions. **d** Scaled expression of SNCA, RHOT1, LRRK2 and GBA genes in IPD and CTRL samples considering the expression across all cells or a subset of dopaminergic neuron clusters (DNs mixed and DNs vulnerable). Dot size represents the percentage of cells expressing the gene, colour represents the gene expression level. Statistical significance was determined using the Wilcoxon rank-sum test. **e** Unsupervised clustering of cell populations within IPD and CTRL samples based on the average cell expression of the 25 genes of interest shared between monogenic PD datasets.

We performed single-cell RNA sequencing on iPSC-derived midbrain organoids at day 50 of organoid culture from six (three female and three male) IPD patients and six (three female and three male) healthy controls (Supplementary Table 10). Using cell type-specific markers^38,39^, we identified nine distinct cellular populations—GABAergic neurons, subdivided into mature and young GABAergic neurons, dopaminergic neurons, subdivided into mixed and vulnerable populations, neuroblasts, radial glia, neuronal stem cells, astrocytes and oligodendrocytes (Fig. 3a and Supplementary Fig. 4) that were all present in IPD and healthy control samples (Fig. 3b). The two dopaminergic neuron clusters were named ‘mixed’ characterised by the expression of A9 dopaminergic neuron markers along with GABAergic and glutamatergic neuron markers (Supplementary Fig. 4) and ‘vulnerable’ according to the expression of dopaminergic neuron vulnerability markers^40^ (Supplementary Fig. 5).

A significant reduction in TH-positive dopaminergic neuron levels was reported in the four datasets of midbrain organoids derived from monogenic PD patient iPSCs, regardless of the pathogenic variant carried by the patients^31,33–35^. Thus, first, we wanted to confirm that IPD midbrain organoids also show a reduction in the dopaminergic neuron populations compared to healthy controls. We assessed the percentage of each cell type in control and IPD conditions (Fig. 3c and Supplementary Fig. 6) and observed that in the IPD case, there is a 1.7 times reduction of mixed dopaminergic neuron population and 1.5 times less vulnerable dopaminergic neurons. Additionally, GABAergic neuron populations and radial glia cells were reduced in IPD compared to control samples. However, the astrocyte, oligodendrocyte, neuroblast and neuronal stem cell populations were slightly larger in IPD than in healthy control midbrain organoids (Fig. 3c and Supplementary Fig. 6).

Further, we checked the expression pattern of PD-associated genes (LRRK2, SNCA, GBA and RHOT1 encoding MIRO1) corresponding to the monogenic PD datasets. We observed that SNCA and RHOT1 expression levels were significantly reduced in all cells, as well as in dopaminergic neurons of IPD samples compared to healthy controls (Fig. 3d). LRRK2 expression was significantly higher in IPD samples taking all cells together but in dopaminergic neurons the expression difference was insignificant. Similarly, GBA was significantly more expressed in IPD samples analysing its bulk expression; however, it showed lower expression in IPD dopaminergic neurons compared to healthy control samples. The significant dysregulation of genes associated with monogenic forms of PD suggests their potential role in IPD development and shared molecular disease mechanisms between IPD and monogenic PD.

To investigate if there is a similar genetic dysregulation between PD monogenic forms and IPD, we inspected the expression level of the 25 genes of interest shared between at least two monogenic PD experiments (Fig. 2a and Supplementary Table 5) in the IPD single-cell RNA sequencing dataset. We observed that the expression pattern of these 25 genes clearly separated the IPD and the healthy control glial populations—radial glia, astrocytes and oligodendrocytes (Fig. 3e).

**Fig. 4:**
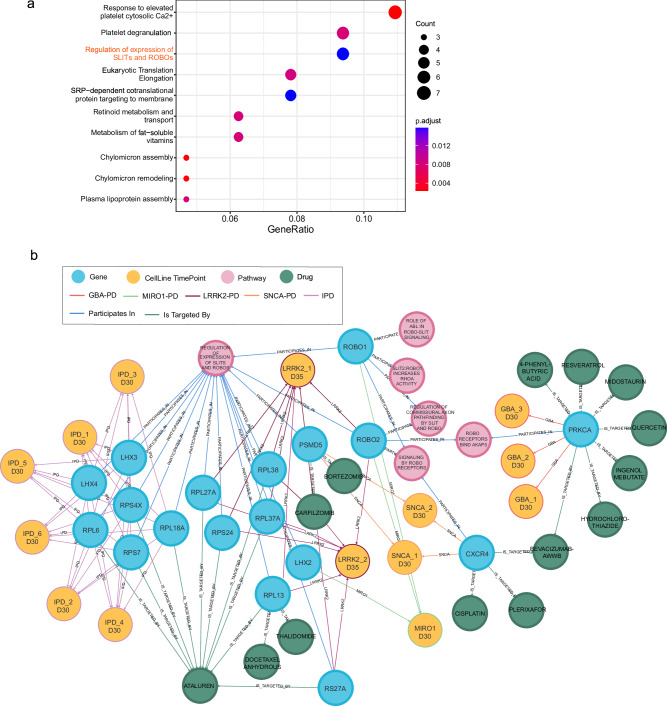
ROBO signalling dysregulation in monogenic and idiopathic PD. **a** Pathway overrepresentation analysis of the top 100 significantly differentially expressed genes between IPD and CTRL samples. Average gene expression across all cells considered for differential expression analysis. ROBO signalling as a re-occurring pathway is highlighted in red. **b** Visual representation of PD-KG subset demonstrating relationships between cell lines (yellow nodes) and genes (blue nodes) involved in pathways associated with ROBO signalling (pink nodes) from four genetic PD and IPD datasets of the top 100 significantly differentially expressed genes with drugs from DGIdb (green nodes). The colour of the edges indicates different datasets (GBA-red, LRRK2-dark purple, MIRO1-green, and SNCA-orange IPD-light purple).

Finally, we determined the significant DEGs between IPD and healthy control midbrain organoids (bulk-mode). In total, there were 6321 significant DEGs (p.adjust < 0.05). ORA analysis showed that the top 100 significant DEGs between IPD and control samples are involved in platelet functions, metabolic processes, and regulation of SLIT/ROBO signalling involved in axon guidance (Fig. 4a). The occurrence of ROBO signalling, which was found already for the monogenic PD cases, among the most enriched pathways based on the top 100 DEGs in IPD, suggests that ROBO signalling might play an important converging role in PD, independently of disease aetiology, providing a potential link between the pathophysiology of monogenic and idiopathic PD. In addition, we performed an ORA analysis on the complete list of DEGs from each individual dataset in order to further validate the commonly dysregulated biological processes across all PD cases (Supplementary Fig. 7). Results revealed that axonogenesis and axon development are altered in all monogenic PD cases (except SNCA-PD), as well as in IPD. Additionally, several pathways related to synapse development and functionality were significantly enriched in both monogenic PD cases and IPD (Supplementary Fig. 7). Both axonal growth and synapse development are regulated by ROBO signalling, further supporting its role in all PD cases described here. Next, we used the PD-KG to explore the genes associated with ROBO signalling in individual datasets (Fig. 4b). A set of 18 genes across four monogenic PD datasets and the IPD dataset showed involvement in ROBO signalling-associated pathways as follows LRRK2 (7 genes), SNCA (2 genes), GBA (one gene), MIRO1 (3 genes) and IPD (6 genes). Only the ROBO2 gene was a common gene among the datasets, present in both LRRK2 and MIRO1 datasets. Details on the presence of these 18 genes in the experimental datasets are given in Supplementary Table 11. Further, we used the PD-KG to identify the drugs targeting genes involved in the ROBO signalling-associated pathways across all datasets. We determined a set of 14 such drugs, including medications used in cancer treatment (Thalidomide, Plerixafor, Midostaurin, Docetaxel Anhydrous, Cisplatin, Carfilzomib, Bortezomib, Bevacizumab-AwwB), treatment of metabolic disorders (4-Phenylbutyric acid) as well diuretic medications (Hydrochlorothiazide), plant-derived chemical compounds with pleiotropic effects (Resveratrol, Quercetin, Ingenol mebutate) and medication used for the treatment of Duchenne muscular dystrophy (Ataluren) (Fig. 4b and Supplementary Table 12). The list of drugs approved for other diseases that target the same genes found to be significantly dysregulated in PD may serve as a data-driven hypothesis for future PD treatment studies.

## Stratification of idiopathic PD

**Fig. 5:**
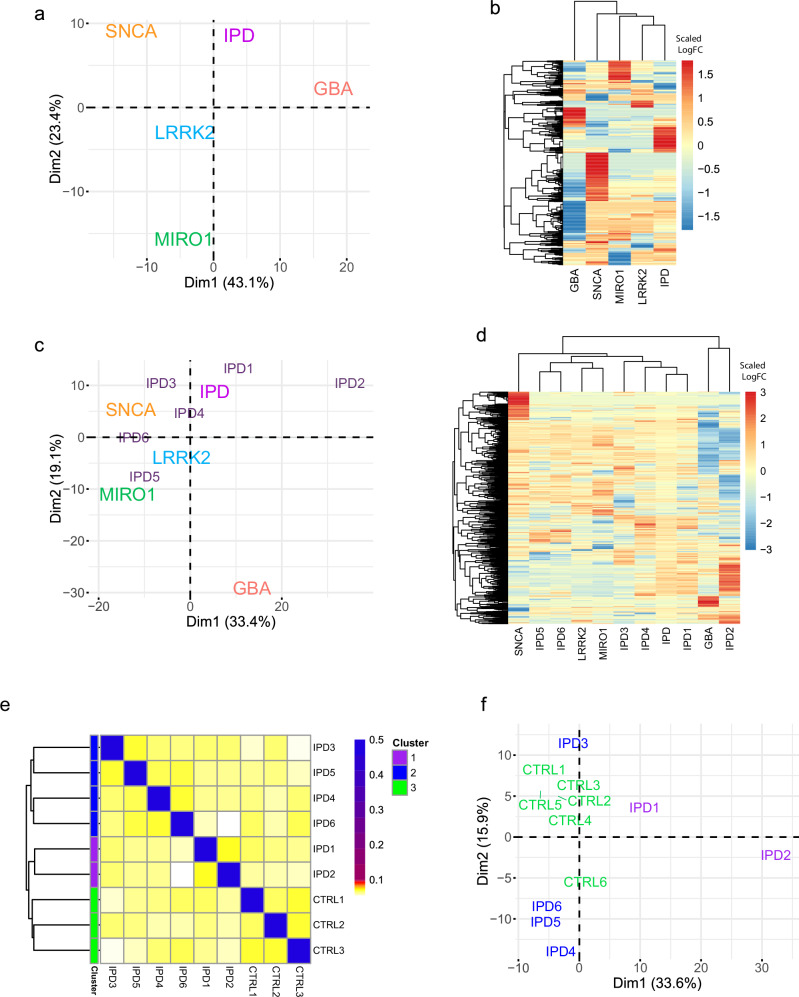
IPD stratification. **a** Two-dimensional PCA plot of the monogenic PD and IPD datasets. **b** Unsupervised clustering of monogenic PD and IPD datasets. **c** Two-dimensional PCA plot of the monogenic PD datasets and IPD datasets, considering every individual IPD sample separately. **d** Unsupervised clustering of monogenic PD and IPD datasets, considering every individual IPD sample separately. **a**–**d** Log2 FC of individual experiments considered (PDvsCTRL) of the genes included in the merged list of the top 100 significantly differentially expressed from each individual experiment. **e** Similarity network fusion analysis on integrated imaging and transcriptomic features (top 100 significantly differentially expressed genes) from 6 IPD and 3 CTRL cell lines. The computed similarity score among different cell lines is within the [0, 0.1] range in this set. The self-similarity has a value of 0.5. The colour palette shows the similarity among the cell lines: “white” for values close to 0, “yellow” for low values within the range [0, 0.1], “red” for values closer to 0.1, and “blue” for self-similarity (0.5). **f** Two-dimensional PCA plot of IPD and CTRL samples, considering the average cell expression of the top 100 significantly differentially expressed genes between every IPD line against all six CTRL cell lines used for single-cell RNA sequencing.

We observed that LRRK2, SNCA, GBA and RHOT1 genes are significantly differentially expressed in IPD midbrain organoids compared to healthy controls (Fig. 3d). This suggests that at least some IPD samples should exhibit similar phenotypes to those associated with a mutation in the respective genes. To see if the IPD transcriptomic signature is similar to monogenic PD, we performed principal component analysis (PCA) and unsupervised hierarchical clustering on the top 100 significant DEGs for each mutation and IPD against healthy controls. Considering the technical variability of sequencing data (bulk or single-cell), for the stratification analysis we used log2 fold change PD vs control, calculated for each dataset separately. The PCA displayed the high heterogeneity of PD, positioning almost every monogenic PD case in a separate quadrant of the PCA plot (Fig. 5a). The IPD and LRRK2-PD were clustered closer to the zero axis, suggesting more similar disease mechanisms. Similarly, in the heatmap of unsupervised hierarchical clustering, we observed that IPD and LRRK2-PD were clustered together, while other PD forms showed distinct transcriptomic signatures (Fig. 5b). When looking at IPD cases individually, we observed that the first two data dimensions explain only about 50% of the variance with the most separation across the first dimension, highlighting the PD complexity (Fig. 5c). In the heatmap, we observed that most IPD samples were rather clustered together, with LRRK2-PD and MIRO1-PD, except IPD2 with a transcriptomic signature more similar to GBA-PD (Fig. 5d).

In addition, we performed a stratification analysis for the individual IPD-patient-specific midbrain organoid cell lines against healthy controls using a similarity fusion network (SNF) approach^41^. The SNF method computes the similarity of samples using multiple types of associated data rather than a single type, and it has been used for patient stratification in various biomedical projects, including the SNF analysis on a de novo PD cohort from Parkinson’s Progression Markers Initiative (PPMI)^42^. Here, we integrated the set of the top 100 significant DEGs and the imaging data of IPD midbrain organoids comprising quantification of 8 core proteins (namely TUBB3, S100B, GFAP, TH, MAP2, PAX6, KI67, SOX2), and TH fragmentation index from 6 IPD cell lines and the 3 healthy control cell lines (Fig. 5e). Of note, here we selected only the 3 healthy control cell lines that were included in both imaging and transcriptomics data as the SNF method requires that the sample set is consistent across the data type layers (all samples are described by all considered data type layers)^41^. The predefined number three of SNF clusters separated control samples from IPD samples, subdividing IPD samples into two groups. One of the IPD clusters included three male (IPD4, IPD5 and IPD6) and one female (IPD3) IPD patient-derived samples (Fig. 5e cluster 2). The second IPD cluster included the other two female (IPD1 and IPD2) IPD patient-specific samples (Fig. 5e cluster 1), suggesting rather sex-independent PD mechanisms. Furthermore, the PCA approach on the transcriptomic signature of the top significant DEGs, including all six control lines, supported the clustering of IPD1 and IPD2 lines by SNF analysis (Fig. 5f). The same IPD1 and IPD2 samples were also separated from other IPD lines and controls in Fig. 5d; IPD2 clustered with GBA-PD and IPD1 being the farthest away from the LRRK2 and MIRO1 clusters. Clustering of IPD3, IPD4, IPD5 and IPD6 closer to LRRK2-PD and MIRO1-PD (Fig. 5c, d), and at the same time closer to healthy controls (Fig. 5f) suggests that they might share disease mechanisms with LRRK2-G2019S and MIRO1-R272Q associated PD.

Finally, we ran a core analysis on gene expression levels in the Ingenuity Pathway Analysis (IPA) platform^43^ to predict the significantly enriched pathways and their activity level for the individual IPD cases (Supplementary Fig. 8). The analysis revealed the molecular heterogeneity of IPD demonstrating a variety of dysregulated metabolic and signalling pathways, including again ROBO signalling, downregulated in one of the IPD cell lines. The predicted activity (activation or inhibition) based on the log2 fold change values often showed an opposite trend for different IPD samples regarding the same pathway; for example, this was the case for protein ubiquitination, synaptogenesis, and mitochondrial dysfunction, again suggesting that the same molecular processes may be differentially regulated in different IPD cases.

Altogether these results show that IPD can be stratified in relation to monogenic PD, which is important for targeted treatment approaches and a step towards personalised medicine.

## Discussion

Contextualisation using already-established knowledge is an important and often forgotten tool that could facilitate biomedical result interpretation. Moreover, secondary data analysis is important in order to make use of previously generated data as such, enhancing the potential of every dataset. Here, we demonstrated an example of experimental data integration with external data sources, enabling better data contextualisation and exploration using previous knowledge. The main limitation was the heterogeneity of the available imaging datasets that did not share most of the quantified features (core proteins). Moreover, the acquisition of the imaging and transcriptomics data was also different in terms of frequency and time points of the collection. Specifically, the image data acquisition was done longitudinally, with different time points across different organoid models. The transcriptomics data was collected at a single time point for each organoid culture (either D30 or D35, depending on the protocol of the initial study, except for IPD organoids, where transcriptomics experiment was done at day 50 as phenotypes in IPD midbrain organoids emerge later than in PD patient organoids carrying a pathogenic variant). Therefore, for the analysis here, we mainly considered imaging data acquired at the closest time point to transcriptomics data. These limitations were also addressed by performing complementary statistical and computational analyses to better explore the input datasets, including the development of the PD-KG (which connects the datasets with public biological repositories and facilitates contextualisation and network-based analysis), the enrichment analysis for all RNA sequencing datasets and SNF stratification for the IPD cell lines. Overall, the identification of these limitations allows us to further develop and optimise our experimental designs and expand the network by including missing imaging data. In the future, the PD-KG can be complemented with transcriptomics data of other time points to explore genotype-phenotype relationships over time. Despite limitations, we were able to find common disease-associated features and tendencies between monogenic and idiopathic forms of PD. In addition, the integration of external data sources into PD-KG allowed the exploration of shared dysregulated pathways and approved drugs targeting dysregulated genes, which indicates the benefits of using network-based approaches for biomedical data exploration, analysis and visualisation, and hypothesis derivation. Additionally, the PD-KG can aid in designing future experiments by comparing core protein expression across different healthy control cell lines at various time points.

Although we did not identify a single DEG shared among the LRRK2-G2019S, 3xSNCA, GBA-N370S, and MIRO1-R272Q datasets, highlighting the heterogeneity of PD, we did identify 25 genes commonly dysregulated in at least two of these datasets. Furthermore, these 25 genes were seen as involved in pathways closely linked to those associated with the unique genes from all four datasets, including IQ motif-containing GTPase-activating proteins (IQGAPs), glycolysis and translocation of glucose transporter GLUT4, as well as pathways involved in the organisation of the cytoskeleton, axonal guidance and neuronal migration, such as ROBO signalling and recycling pathway of L1 and pathways regulating neuronal connections and communication such as EPH-ephrin mediated repulsion of cells and synaptic transmission. These results indicate that distinct PD transcriptomic signatures might still lead to dysregulation of the same molecular mechanisms. Interestingly, several of the 25 genes were cytoskeleton proteins—ACTB, ACTG, TUBB2B, and TUBA1A. Cytoskeleton filaments are crucial not only during developmental stages ensuring the neuronal and glial morphological complexity but also play a crucial role in mature neurons regulating such important cellular functions as axonal transport, the transmission of electric and chemical signals and ensuring resilience against stress or aging-associated processes^44^. Furthermore, tubulin rearrangement has been observed in the *substantia nigra* of PD patients, also correlating with α-Synuclein oligomerization and decreased axonal compartment^45^. Whereas differential expression of tubulins (TUBB2A, TUBA1A, TUBB, TUBA1B) has been associated with stress responses in iPSC-derived dopaminergic neurons carrying SNCA A53T mutation^46^. In addition, several of these 25 genes, namely CHL1, and ROBO2, are known to regulate axonal guidance, growth and synaptogenesis. For example, CHL1 expression has been shown to be significantly downregulated iPSC model of PRKN deletion^47^, while significant dysregulation of ROBO2 has been found in IPD patient post-mortem samples^48,49^.

In line with that, our results showed ROBO signalling among the top most enriched pathways of the significant DEGs between IPD and healthy control midbrain organoids. ROBO signalling regulates neuron migration, and axonal guidance, and is also involved in the control of the balance between cell proliferation and differentiation^37^. Studies in mice have shown that ROBO signalling is involved in the regulation of dopaminergic neuron projections^50^. In addition, dopaminergic neurons derived from human embryonic stem cells show ROBO2 protein expression increase over time and display robust response to axonal guidance cues by SLIT2, which is regulated by ROBO2^51^. Additionally, downstream effects of the SLIT/ROBO signalling are associated with microtubule cytoskeleton organisation, including GTPase regulation^52^. Both MIRO1 and LRRK2 have GTPase domains and are direct regulators of actin cytoskeleton dynamics^53–55^. Accordingly, cytoskeleton filaments actin (ACTB) and different subtypes of tubulins (TUBB3, TUBA1A, TUBB2B) were among the top 100 significant DEGs in the LRRK2 and MIRO1 PD datasets. The common dysregulation of cytoskeleton dynamics might link LRRK2-G2019S and MIRO1-R272Q-associated PD. Notably, the majority of the 25 shared DEGs were between these two PD datasets, and they were positioned closer in the clustering analysis. However, we could identify significant DEGs associated with the SLIT-ROBO pathway also in other monogenic PD datasets and in the IPD dataset (Fig. 4b), suggesting that SLIT/ROBO might play an important role in PD independently of disease aetiology, providing a potential link between the pathophysiology of monogenic and idiopathic PD. In the PD-KG we could see that multiple of the genes involved in the regulation of SLIT proteins were ribosomal, indicating potential aberrant protein translation affecting SLIT protein functionality. Interestingly, the drug Ataluren, targeting 4 out of the 6 IPD genes linked to SLIT proteins: RPL18A, RPL6, RPS7 and RPS4X, acts by interacting with the ribosomes, and recruiting near-cognate transfer-RNAs to ensure the full-length-protein synthesis from messenger-RNA, surpassing the nonsense mutations^56^. Missense mutations account for a large proportion of human diseases, and currently, RNA targeting therapies are among the successful approaches for treating genetic disorders caused by missense mutations^57^. It is known that PD is a multifactorial disorder and genetics together with environment and age play a role in the disease pathophysiology^3^. Thus, it is feasible that there are missense mutations that contribute to the development of PD cases classified as idiopathic. Additionally, the PD-KG allowed us to identify several other existing medications targeting dysregulated, SLIT/ROBO signalling-associated genes across all included datasets. Importantly, drug-gene network analysis can be also expanded to other dysregulated genes, highlighting the practical value of the PD-KG in data contextualisation and the generation of new hypotheses, which could be valuable for future PD drug screening studies.

Interestingly, our analysis revealed that radial glia, astrocyte, and oligodendrocyte populations were discriminated between IPD and control samples based on the 25 gene expression with shared dysregulation between monogenic PD cases. This suggests that some glial properties or functions could be compromised in IPD. Among other things, glial cells are known to provide metabolic support to neurons. It has been suggested that in neurodegenerative diseases, glial cells undergo metabolic changes that enhance neuroinflammatory responses while reducing their neuroprotective and supportive functions^58^. Accordingly, ORA indicated that several metabolism-related pathways were among the enriched ones based on the top 100 significant DEG sets. These metabolic alterations may be a result of the inflammatory phenotype driving glial metabolic dysfunction, which in turn contributes to neuronal metabolic changes. Further investigation of the expression of these 25 genes at the single-cell level in monogenic PD is needed to determine whether the altered glial cell transcriptomic profile indeed represents a common mechanism in PD.

Aiming to stratify IPD, we extended the data exploration and analysis from the PD-KG by using a set of methods and tools (including ORA, unsupervised hierarchical clustering, PCA and SNF) that provided complementary details on the upper biological levels (enriched pathways or biological processes and functions) rather than on single molecules (dysregulated protein or gene) across the input datasets. Moreover, the results from these analyses were obtained from quantitative information (such as transcriptomics or imaging data from the IPD dataset, log2 fold change between IPD samples and healthy controls) rather than on descriptive information integrated into the PD-KG (such as protein-pathway involvement). First, the ORA results on the enriched pathways for the monogenic and IPD datasets were in agreement with the PD-KG results on the pathways shared across molecules of interest. The SNF analysis, which focused on exploring the similarities and differences within the IPD dataset by integrating the two layers of data available, specifically the cell-line-specific imaging data and the transcriptomics, indicated a separation between the control vs IPD samples. The SNF analysis also anticipated subtype identification across the IPD cell lines, which we also could observe in the PCA. Although the stratification of IPD holds great promise in the context of personalised medicine and targeted clinical studies, the main limitation here was the relatively low number of cell lines available, as well as the relatively low number of features considered within each data layer, especially in the imaging data.

Finally, the current version of the PD-KG can be enhanced through the integration of additional types of data and their relationships, including experimental data such as metabolomics and proteomics, as well as open-source resources such as the Human Protein Atlas^59^. This integration would increase the relevance and robustness of the generated hypotheses. The PD-KG can be also enriched with midbrain organoid data from PD patients carrying other PD-associated mutations. Given that midbrain organoids rather represent a developing brain at embryonic stages and therefore primarily reflect early disease mechanisms, additional integration of published data from PD patients potentially would be of further importance. This integration would enable the exploration of shared molecular mechanisms across different forms of PD, particularly those influenced by ageing.

**Fig. 6:**
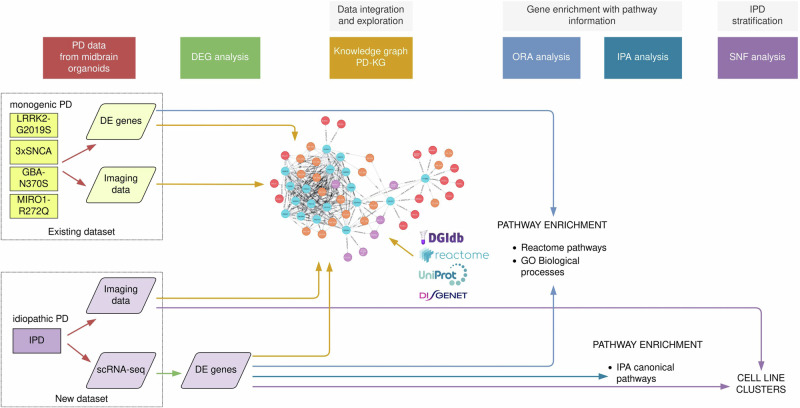
Schematic representation of our workflow. Workflow included the harmonisation, normalisation and integration of multiple monogenic PD datasets, the generation of the IPD dataset, the development of the PD-KB based on data from experimental studies and public repositories, and the application of several statistical and computational methods (network-based analysis and pathway enrichment) towards IPD stratification. The colour of the arrows represents different methods applied in this study.

In conclusion, our study presents a comprehensive multimodal data integration and analysis approach for PD organoids (Fig. 6). Importantly, using the created PD-KG we were able to derive a hypothesis of altered axonogenesis and dysregulated ROBO signalling as common disease mechanisms between the monogenic PD forms included in this study, which was further observed in the newly generated IPD single-cell RNA sequencing dataset.

## Methods

Our workflow consisted of several complementary major steps within the systems of biomedicine. This included harmonising and normalising multiple existing monogenic PD datasets and integrating them into the PD-KG, along with data from public biological repositories. We also generated the IPD dataset and applied statistical and computational methods for network-based analysis and pathway enrichment to help with IPD stratification. A summary of our methods is shown in Fig. 6.

### Ethics approval

Written informed consent was obtained from all individuals who donated samples to this study. The work with iPSCs has been approved by the Ethics Review Panel (ERP) of the University of Luxembourg and the national Luxembourgish Research Ethics Committee (CNER, Comité National d’Ethique de Recherche) under the approval number CNER No. 201901/01 (ivPD).

### Midbrain organoid culture

Cell lines used in this study are summarised in Supplementary Table 10. iPSCs were obtained from six healthy individuals (three female and three male individuals) and six age IPD patients (three female and three male individuals). Neuroepithelial stem cells (NESCs) derivation from iPSCs was performed as described in Reinhardt et al.,^60^ via embryoid body formation and expansion of neuroectoderm. Midbrain organoids were generated and cultured as initially described in Monzel et al.,^61^ (for image analysis) and further optimised by Nickels et al.,^62^ (for RNA sequencing). In brief, NESCs at 80% of confluency were detached using Accutase (Sigma, Cat# A6964). Live cells were counted using Trypan Blue. The 9 × 10^5^ cells were collected into 15 ml of N2B27 maintenance media—DMEM-F12 (Thermo Fisher Scientific, cat.no 21331046) and Neurobasal (Thermo Fisher Scientific, Cat# 10888022) 50:50, supplemented with 1:200 N2 supplement (Thermo Fisher Scientific Cat# 17502001), 1:100 B27 supplement w/o Vitamin A (Life Technologies, Cat# 12587001), 1% GlutaMAX (Thermo Fisher Scientific, Cat# 35050061) and 1% penicillin/streptomycin (Thermo Fisher Scientific, Cat# 15140122). Cells were distributed in 96-well ultra-low attachment plates (faCellitate, Cat# F202003)—150 µl with 9000 cells per well. Plates were centrifuged at 300×*g* for 1 min to facilitate spheroid formation at the bottom of the well. On the 2nd day of organoid culture, media was changed to N2B27 patterning media, which is N2B27 base media supplemented with 10 ng/ml hBDNF (Peprotech, Cat# 450-02-1 mg), 10 ng/ml hGDNF (Peprotech, Cat# 450-10-1 mg), 500 µM dbcAMP (STEMCELL Technologies, Cat# 100-0244), 200 µM ascorbic acid (Sigma), 1 ng/ml TGF-β3 (Peprotech Cat# 100-36E) and 1 µM purmorphamine (Enzo Life Science, Cat# ALX-420-045). The next media change was done on the 5th day of organoid culture. On day 8 of organoid culture, organoids for high-content imaging were embedded in extracellular matrix-like Geltrex (Thermo Fisher Scientific, Cat# A1413302) droplets^63^. Embedded organoids were kept in dynamic conditions on an orbital shaker (IKA), rotating at 80 rpm until the collection day. From day 8 of organoid culture organoids were kept in N2B27 differentiation media which only differs from the patterning media by lacking purmorphamine. Media changes were done every 3–4 days for embedded and non-embedded organoids until the day of sample collection. For single-cell sequencing, organoids were collected at day 50. For imaging, organoids were collected at days 30, 60, 90 and 180 of organoid culture. Cell culture was regularly tested for mycoplasma contamination using LookOut® Mycoplasma PCR Detection Kit (Sigma, Cat# MP0035-1KT).

### Immunofluorescence staining of organoid sections

Midbrain organoids were collected in a new 24-well plate (one organoid per well) and fixed with 4% paraformaldehyde (PFA) for 6-7  h at room temperature (RT) followed by washing three times with PBS for 15 min. Individual midbrain organoids were then embedded in 3% low-melting point agarose (Biozym Scientific GmbH, Cat# 840100). Midbrain organoids were sliced into 80 µm sections using a vibrating blade microtome (Leica VT1000s). Selected sections (per organoid one central section closer to the organoid core and one border section closer to the edge) for immunostaining were incubated for 30 min in 0.5% Triton X-100 at RT on an orbital shaker to permeabilize the cell membrane. Sections then were blocked for 2 h at RT on an orbital shaker with a blocking buffer (2.5% normal donkey serum, 2.5% BSA, 0.01% Triton X-100 and 0.1% sodium azide). Primary antibodies were diluted in the blocking buffer and incubated with sections for 48 h at 4 °C on an orbital shaker. Primary antibodies were stem cell markers PAX6 1:300 (Biolegend #901302, RRID: AB_2749901), SOX2 1:200 (R&D Systems Cat#BAF2018, RRID: AB_356217), KI67 1:200 (BD Biosciences Cat#550609, RRID: AB_393778), neuronal markers MAP2 1:1000 (Abcam Cat#ab5392, RRID: AB_2138153), TH 1:1000 (Abcam Cat#ab112, RRID: AB_297840), TUJ1 1:1000 (BioLegend Cat#802001, RRID: AB_2564645 or Sigma-Aldrich Cat#AB9354, RRID:AB_570918), astrocyte markers S100beta 1:1000 (Sigma-Aldrich Cat#S2532, RRID: AB_477499), GFAP 1:1000 (Millipore Cat# AB5541, RRID:AB_177521) and alpha-Synclein 1:1000 (Novus Cat#NBP1-05194, RRID:AB_1555287). Then sections were washed three times for 5 min with 0.01% Triton X-100 in PBS followed by a 2-h incubation at RT on an orbital shaker, protected from light with the Alexa Fluor^®^ conjugated secondary antibodies and nuclei stain Hoechst 33342 (Invitrogen Cat# 62249) diluted 1:1000 and 1:10 000 respectively in the blocking buffer. Sections were then washed three times for 10 min with 0.01% Triton X-100 in PBS and once with MiliQ water. For imaging sections were mounted on slides (De Beer Medicals, Cat# BM-9244) and covered with mounting media Fluoromount-G® (SouthernBiotech, Cat# 0100-01) and coverslip (VWR, Cat# ECN631-1574).

### Image acquisition and analysis

High-content imaging was performed using the Operetta high-content screening microscope (PerkinElmer) with a 20× objective using Z-stack acquisition selecting 25 planes per section. Acquired images were analysed with a customised pipeline using Matlab (v.2017a, Mathworks, RRID: SCR_001622) as described in ref. ^64^ and ref. ^65^. Only normalised features of the total nuclei count of the analysed organoids were considered in this study.

### Single-cell RNA sequencing and data analysis of IPD dataset

Midbrain organoids were collected from their culture medium and washed with 1× PBS (phosphate-buffered saline, Gibco, Cat#10010-015). Organoids were transferred to a 1.5 ml Eppendorf tube and digested in 1 ml sCelLive™ Tissue Dissociation Solution (Singleron Biotechnologies, Cat#1190062) diluted 1:2 with PBS. The organoids were placed in a thermal shaker at 750 rpm at 37 °C for 45 min. The state of dissociation was checked at regular intervals under a light microscope. Following digestion, the suspension was filtered using a 40-µm sterile strainer (Greiner, Cat#542040). The cells were centrifuged at 350 × *g* for 5 min at 4 °C, and the cell pellets were resuspended in 300 µl PBS. Cells were stained with Acridine Orange/Propidium Iodide Stain (Logos Biosystems, Cat#F23001), and the cell number and viability were calculated using LUNA-FX7™ Automated Cell Counter (Logos Biosystems, Villeneuve d’Ascq, France).

The single-cell RNA-seq libraries were constructed using GEXSCOPE™ Single Cell RNAseq Library Kit (Singleron Biotechnologies, Cat#4180011) according to the manufacturer´s instructions.

Briefly, for each library, the concentration of the single-cell suspension was adjusted to 3 × 10^5^ cells/ml with PBS, and the suspension was loaded onto an SD microfluidic chip to capture 6000 cells. Paramagnetic beads conjugated to oligodT probes that carry a unique molecular identifier (UMI) and a barcode unique to each bead (from the same kit) were loaded, after which the cells were lysed. The beads bound to polyadenylated mRNA were extracted from the chip and reverse transcribed into cDNA at 42 °C for 1.5 h, and the cDNA was amplified by PCR. The cDNA was then fragmented and ligated to indexed Illumina adaptors. The fragment size distribution of the final amplified library was obtained on an Agilent TapeStation.

The library concentration was calculated using the Qubit 4.0 fluorometer and the libraries were pooled in an equimolar fashion. The single-cell libraries were sequenced on an Illumina NovaSeq X using a 2 × 150-bp approach to a final depth of 90 GB per library. The reads were demultiplexed according to the multiplexing index sequencing on Illumina’s BaseCloud platform.

The pre-processing of the fastq files was conducted using CeleScopeÂ® (v.1.14.1; www.github.com/singleron-RD/CeleScope; Singleron Biotechnologies GmbH) to generate gene count matrices with default parameters. Low-quality reads were removed. Sequences were mapped using STAR (https://github.com/alexdobin/STAR) to the human genome reference version GRCh38 and genes were annotated using Ensembl 92. The reads were assigned to genes using featureCount (https://subread.sourceforge.net/) and the cell calling was performed by fitting a negative bimodal distribution and determining the threshold between empty wells and cell-associated wells to generate a count matrix file containing the number of unique molecular identifiers (UMI) for each gene within each cell.

Downstream analysis was done using Seurat single-cell analysis toolkit (v. 5.0.1; RRID: SCR_016341)^66^ in R computing language (v. 4.3.2; RRID: SCR_001905). Cells with less than 500 genes and more than 6000–7500 (customised setting depending on the sample) were excluded to remove non-viable or low-quality cells and doublets, respectively. In addition, cells having a mitochondrial gene content above 15–20% were also excluded as non-viable cells. Datasets of single cell lines were merged, log normalised and scaled to all genes. The integration was performed using Seurat integration workflow 5.0^66^ based on the first 20 PCA components. Cell populations were identified by applying the Louvain algorithm modularity optimization with a resolution of 0.2^67^. Nine distinct cell clusters were identified and visualised using the uniform manifold approximation and projection (UMAP) technique^68^. Marker genes of each cell population were determined by applying the *FindAllMarkers* function of Seurat. Additionally, cellular identities were validated using the GeneAnalytics online tool^69^ (https://geneanalytics.genecards.org/), PangloaDB^39^ (https://panglaodb.se/) and human midbrain cell type-specific markers reported in La Manno et al.^38^.

### PD data corpus creation from existing PD datasets

First, we created a core data corpus considering the top 100 most significant DEGs (selected by the adjusted p.value from available bulk or single cell-RNA sequencing data) and 12 core proteins selected from high-content imaging data of four independent published datasets of midbrain organoids (Supplementary Table 8). Long-non-coding RNAs and pseudogenes were excluded. The original RNA sequencing experiments were bulk for the GBA-PD and SNCA-PD and single-cell for MIRO1-PD and LRRK2-PD datasets. In the latter case, the top 100 significant DEGs (selected by p.adjusted value) were determined in a bulk mode to enable comparison to the GBA-PD and SNCA-PD RNA sequencing results. For PCA and unsupervised hierarchical clustering on different PD datasets, log2 fold changes of PD vs control samples from every individual RNA sequencing experiment were considered. For PCA on IPD vs control samples from the same dataset, transcriptomics data was integrated as the average gene expression across all cells. Stratification analysis was performed in R (v. 4.3.2; RRID: SCR_001905). Imaging datasets from four previously published studies were harmonised defining common column names. Individual files from each data set were then aggregated based on harmonised conditions, cell line identifier and timepoint. They were then normalised based on mean value of each feature across unique cell lines, and merged into a new dataset file to enable downstream analysis. Normalisation and aggregation were done using Python 3.12 and the pandas package.

Following the acquisition of the IPD dataset, we further extended the list of DEGs corresponding initially to the four studies by integrating the top 100 most significant DEGs (bulk-mode) from the IPD experiment. Similarly, the list of 12 core proteins from the four existing experiments was extended to include the core proteins from the IPD experiments. Following the removal of duplicates, we aggregated a list of 440 unique DEGs and a set of 13 unique core proteins from all five experiments (see Supplementary Table 1).

### PD-KG development

We developed the PD-KG by using the neo4j technology (neo4j-community-4.4.25 version) and the Java Eclipse environment (with Java SE 1.8). At the core, the PD-KB integrates molecular features from high-content imaging and transcriptomics data from PD iPSCs-generated midbrain organoids. We focused on the contextualisation of these molecular features from the experimental datasets. We integrated information on several types of biological relationships involving these molecular features and other biological entities from public biological repositories such as (i) pathway involvement from Reactome (filtered on the human species)^26^, (ii) disease association from DisGeNet^28^ (we filtered on diseases related to central nervous system), (iii) drug targets from DGIdb^29^, (iv) protein-protein association from IntAct (we selected several types of interactions, including association, physical association, colocalization and proximity)^27^. The mapping between the HGNC gene symbols and unique UniProt identifiers was done using UniProtKB^30^. The reference date for data integration was 04/06/2024. In the underlying graph, the data types such as core proteins, genes, disease, drugs, and pathways were represented by nodes, and their inter-relationships were shown as connecting edges. The graph data model is shown in Fig. 1 and the types of data and their inter-relationships are given in Supplementary Tables 1 and 2.

### Similarity network fusion (SNF) for the IPD dataset

We performed an SNF analysis^41^ towards the IPD cell line stratification by integrating imaging data and top 100 transcriptomics from 6 IPD cell lines and 3 healthy controls, respectively. The transcriptomics data were integrated as the average gene expression across all cells. The code was developed in RStudio (version 2024.04.2 + 764) under the following settings: R version 4.1.0 (2021-05-18), Platform: x86_64-apple-darwin17.0 (64-bit), running under: macOS 14.5. We used the SNFtool package for the SNF and the spectralClustering methods, and the pheatmap package for the heatmap of the results.

### Over-representation analysis (ORA) for the monogenic and IPD datasets

We performed an over-representation analysis (ORA)^36^ in R version 4.1.0 (2021-05-18) for the top 100 transcriptomics features selected from the existing monogenic PD and IPD datasets, respectively. We checked the enriched sets of Reactome pathways corresponding to each of the input gene datasets. For a more systematic exploration, we also performed study-specific ORA analysis using the complete list of DEGs (p.adjusted < 0.05) from each dataset, focusing on the enriched GO biological processes. We used the following methods and packages in RStudio, (with the same settings as for the SNF analysis):

- enrichPathway and ReactomePA for the Reactome pathway enrichment;
- enrichGO for the GO biological process enrichment;
- org.Hs.eg.db and AnnotationDbi::select for the gene ID conversion (between the gene symbol and Entrez id);
- dotplot for the visualisation of the enrichment results;
- ggtitle, theme and ggplot2 for the final customised plots of the enrichment results.

### Core analysis using gene expression for pathway activity prediction

We computed the set of top 100 DEG for every IPD cell line in comparison with the healthy samples and performed a core analysis in the Ingenuity Pathway Analysis platform (IPA)^43^ to predict the list of canonical pathways being activated or inhibited in the IPD samples. We used IPA Version 01-21-02, with the following settings for the core analysis: species = human, Reference set = “Ingenuity Knowledge Base (Gene Only”), Relationships to consider = “Direct Relationships”.

## Supplementary information

Supplementary information

## Data Availability

All data supporting the conclusions of this manuscript are publicly available under this DOI: 10.17881/xs23-rk90. RNA sequencing datasets are available on Gene Expression Omnibus (GEO) under the accession codes: GSE237133 (MIRO1-PD), GSE133894 (LRRK2-PD), GSE208784 (GBA-PD), GSE278265 (SNCA-PD), and GSE276684 (IPD).

## Code availability

All computational scripts used for the modelling and analysis are available on GitLab at: https://gitlab.com/uniluxembourg/lcsb/developmental-and-cellular-biology/zagare_tandem_2024.
