# Supplementary material for: Deciphering shared molecular dysregulation across Parkinson’s disease variants using a multi-modal network-based data integration and analysis
Source: NPJ Parkinsons Dis. 2025 Mar 31;11:63. doi: 10.1038/s41531-025-00914-3 (PMC11958823; doi:10.1038/s41531-025-00914-3)
Supplement: Supplementary file 1 — Supplementary information [file 41531_2025_914_MOESM1_ESM.pdf]

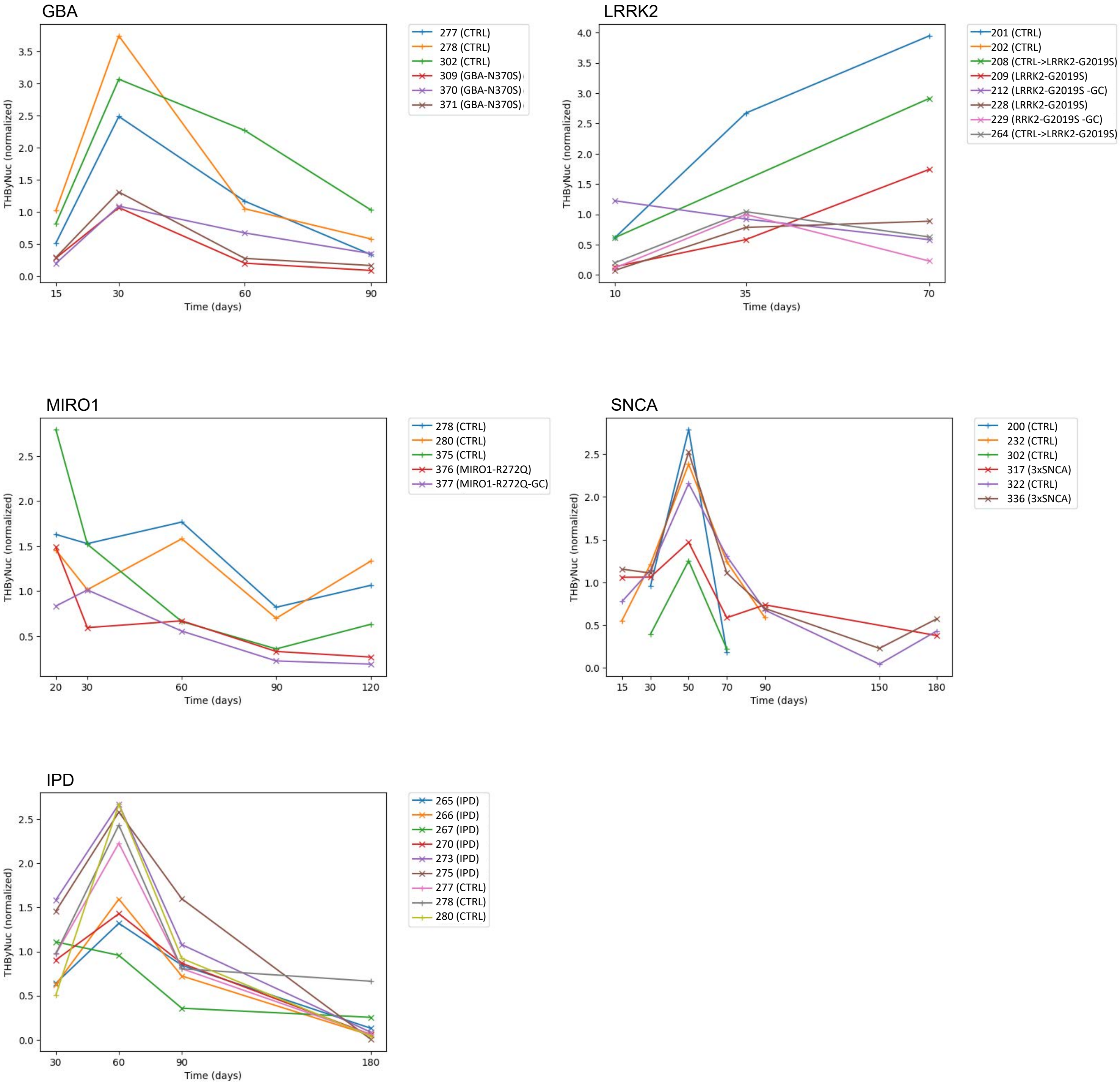

Supplementary Figure 1. Tyrosine hydroxilase expression levels from the high-content imaging data in every individual dataset. Each color represents a cell line from the respective study (for more details on the cell lines see Supplementary Table 3).

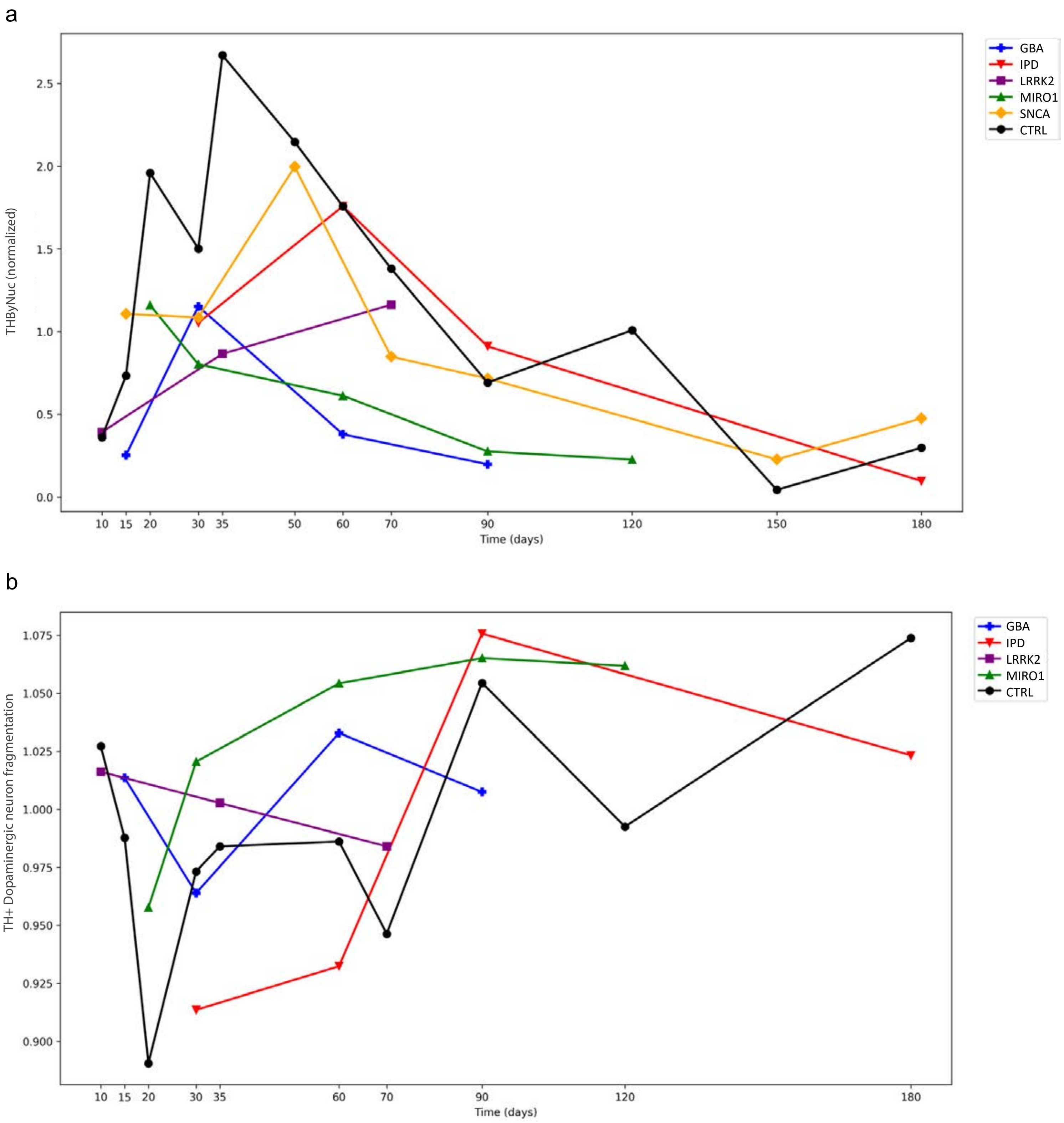

Supplementary Figure 2. Tyrosine hydroxilase (TH) positive dopaminergic neuron a) abundance and b) fragmentation index from the high-content imaging data of every individual dataset aggregated per condition (PD mutation or CTRL). Each color represents a condition (for more details on the cell lines see Supplementary Table 3).

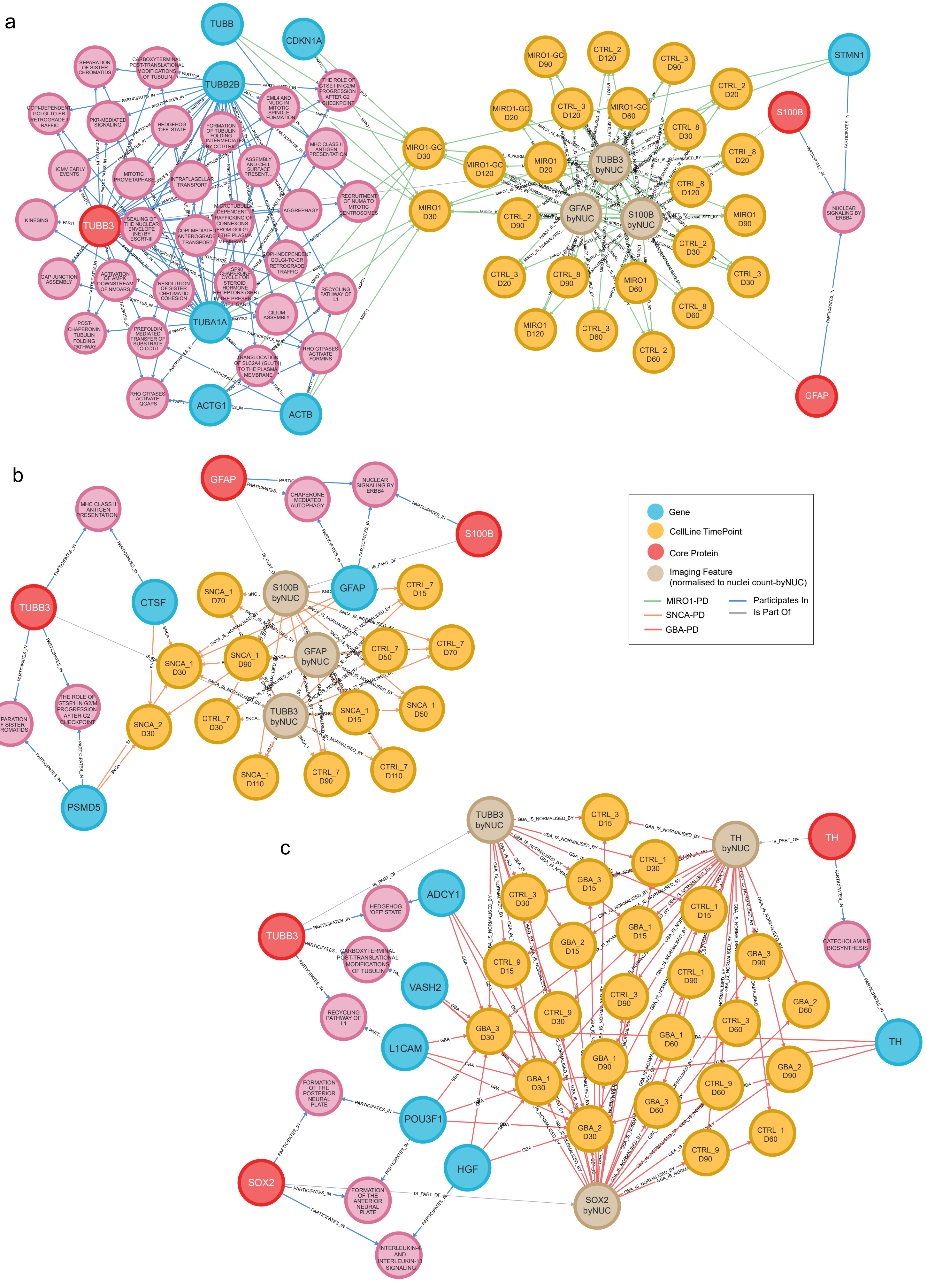

Supplementary Figure 3. Relationships between core proteins and the top significantly differentially expressed genes in individual datasets a) MIRO1-PD, b) SNCA-PD and c) GBA-PD.

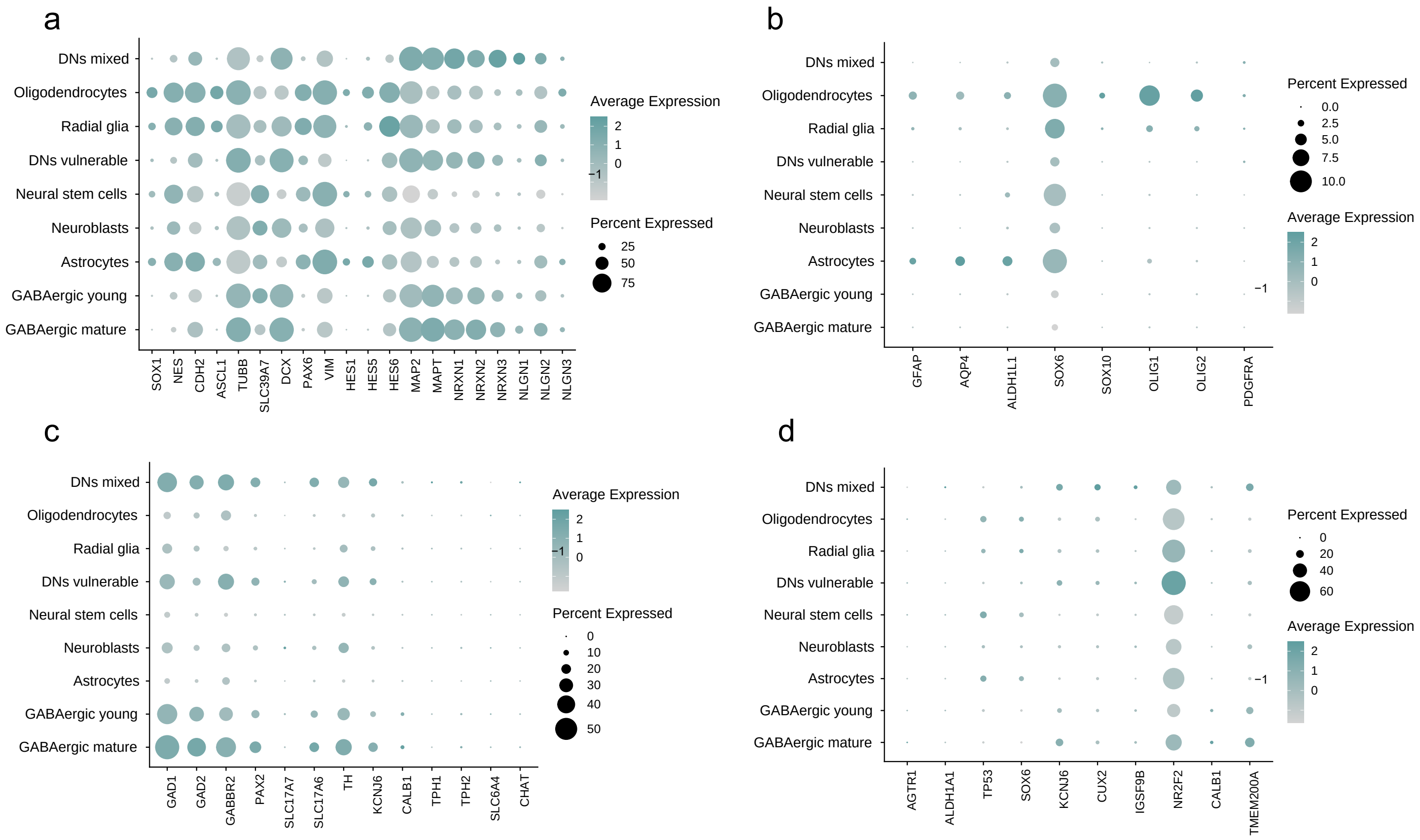

Supplementary Figure 4. Cell type marker expression. a) Neuronal stem cell (SOX1, NES), neuroblast (CDH2, ASCL1, TUBB,SLC39A7, DCX, PAX6), radial glia (VIM, HES1, HES5, HES6) and mature neuron markers (MAP2, MAPT, NRXN1, NRXN2, NRXN3, NLGN1, NLGN2, NLGN3). b) Astrocyte (GFAP, AQP4, ALDH1L1, SOX6) and oligodendrocyte (SOX6, SOX10, OLIG1, OLIG2) markers. Oligodendrocyte progenitor cell marker PDGFRA to confirm oligodendrocyte maturity. c) Neuron subtype markers: GABAergic neurons (GAD1, GAD2, GABBRA2), glutamatergic neurons (PAX2, SLC17A7, SLC17A6), dopaminergic neurons (TH, KCNJ6, CALB1), serotonergic neurons (TPH1, TPH2, SLC6A4) and cholinergic neurons (CHAT). d) Dopaminergic neurons vulnerability (AGTR1, ALDH1A1, TP53, SOX6, KCNJ6, CUX2, IGSF9B, NRF2F2) and resistance (CALB1, TMEM200A) markers.

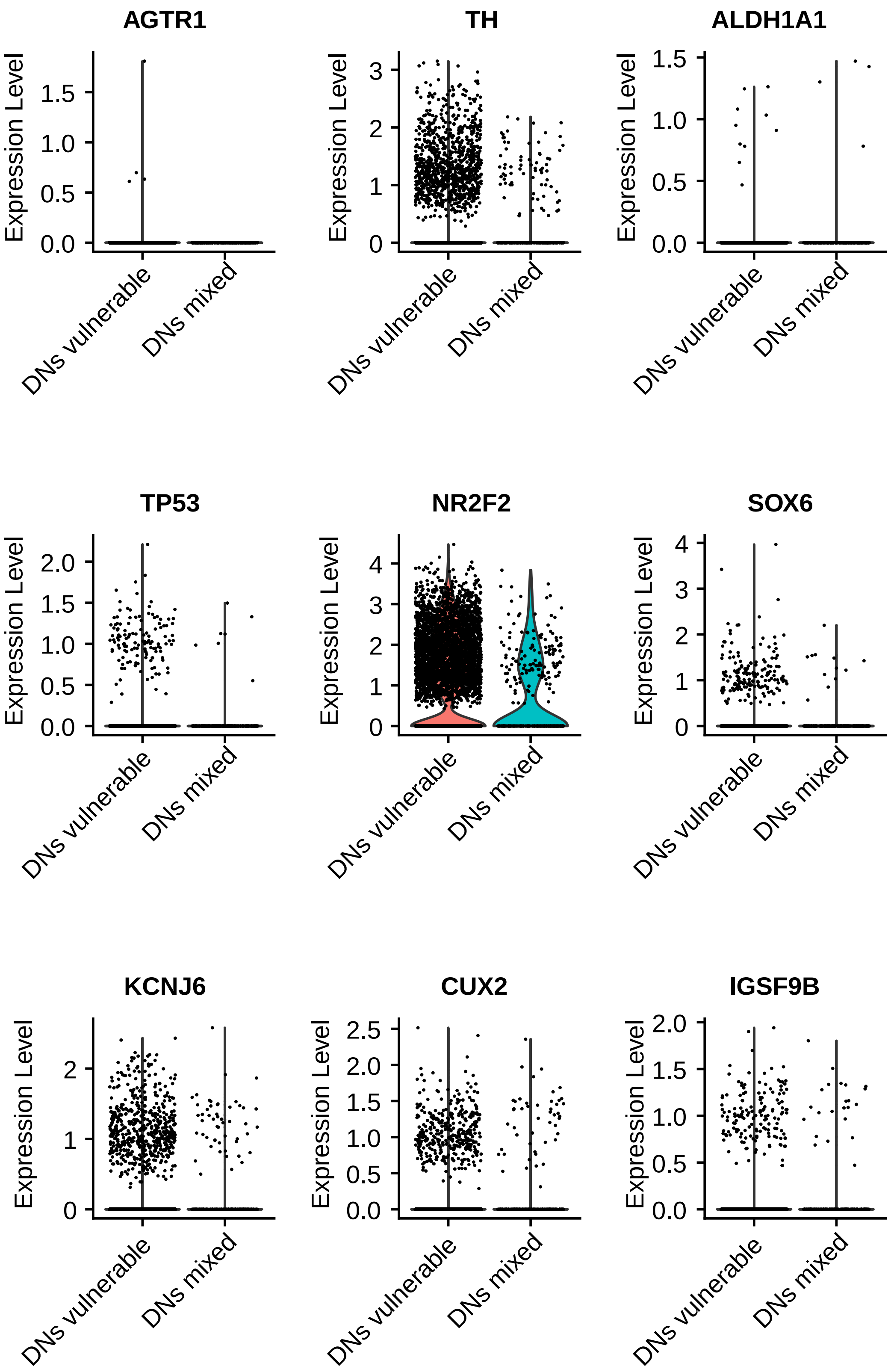

Supplementary Figure 5. Expression of dopaminergic neuron vulnerability markers in the two dopaminergic neuron populations.

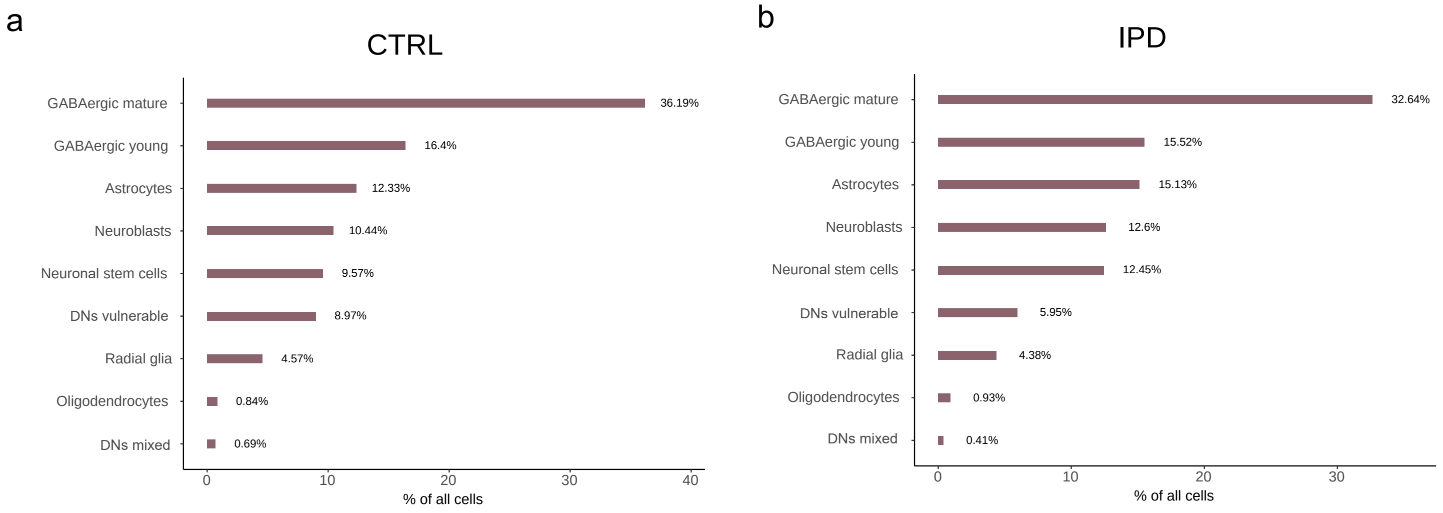

Supplementary Figure 6. Percentage of all cellular populations calculated from the total amount of cells in CTRL and IPD samples.

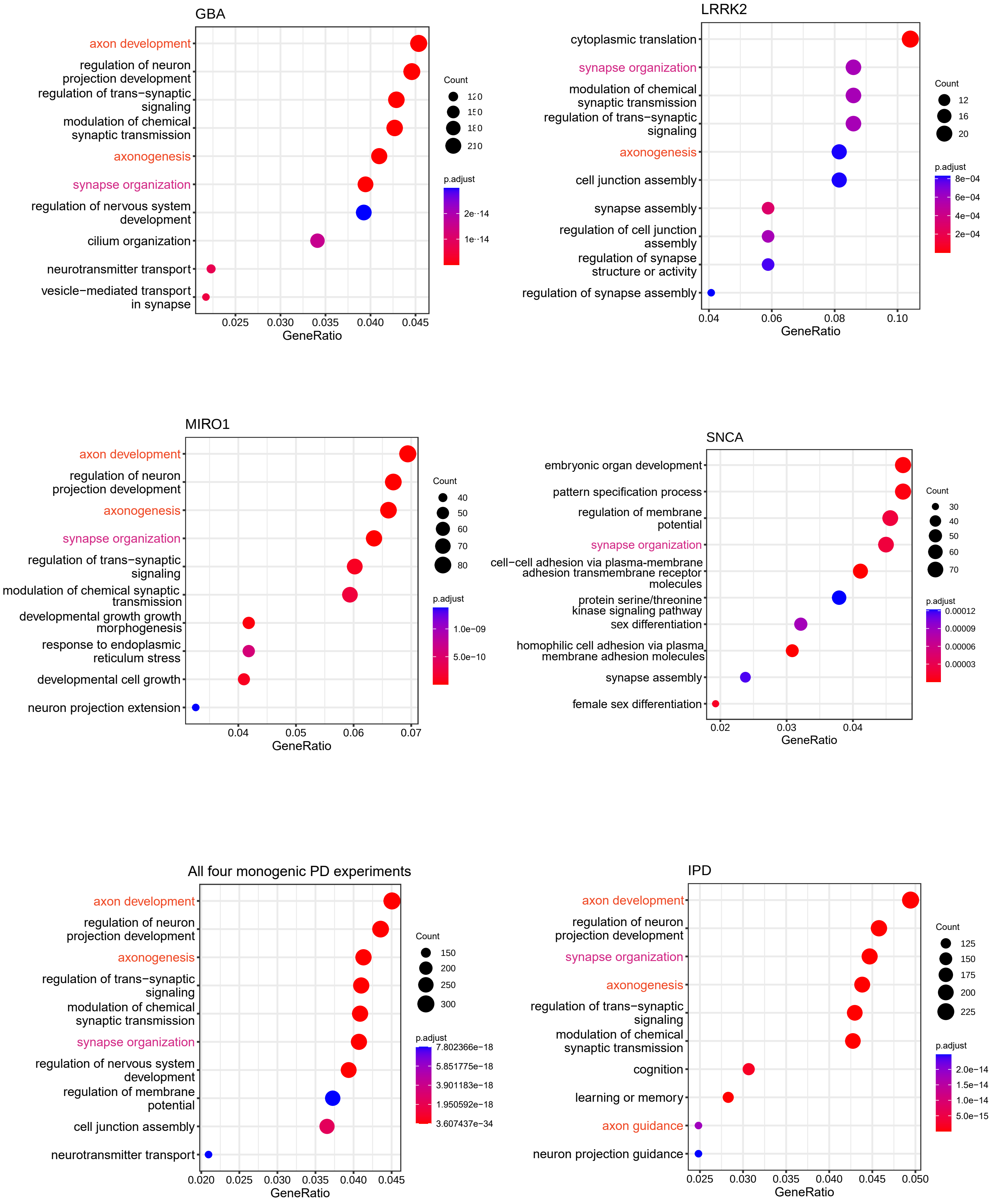

Supplementary Figure 7. Pathway over-representation analysis of biological processes using complete significantly differentially expressed gene list (p.adjust.<0.05) from each individual study and on merged list across all four monogenic PD datasets. Pathways identified as common across datasets are highlighted.

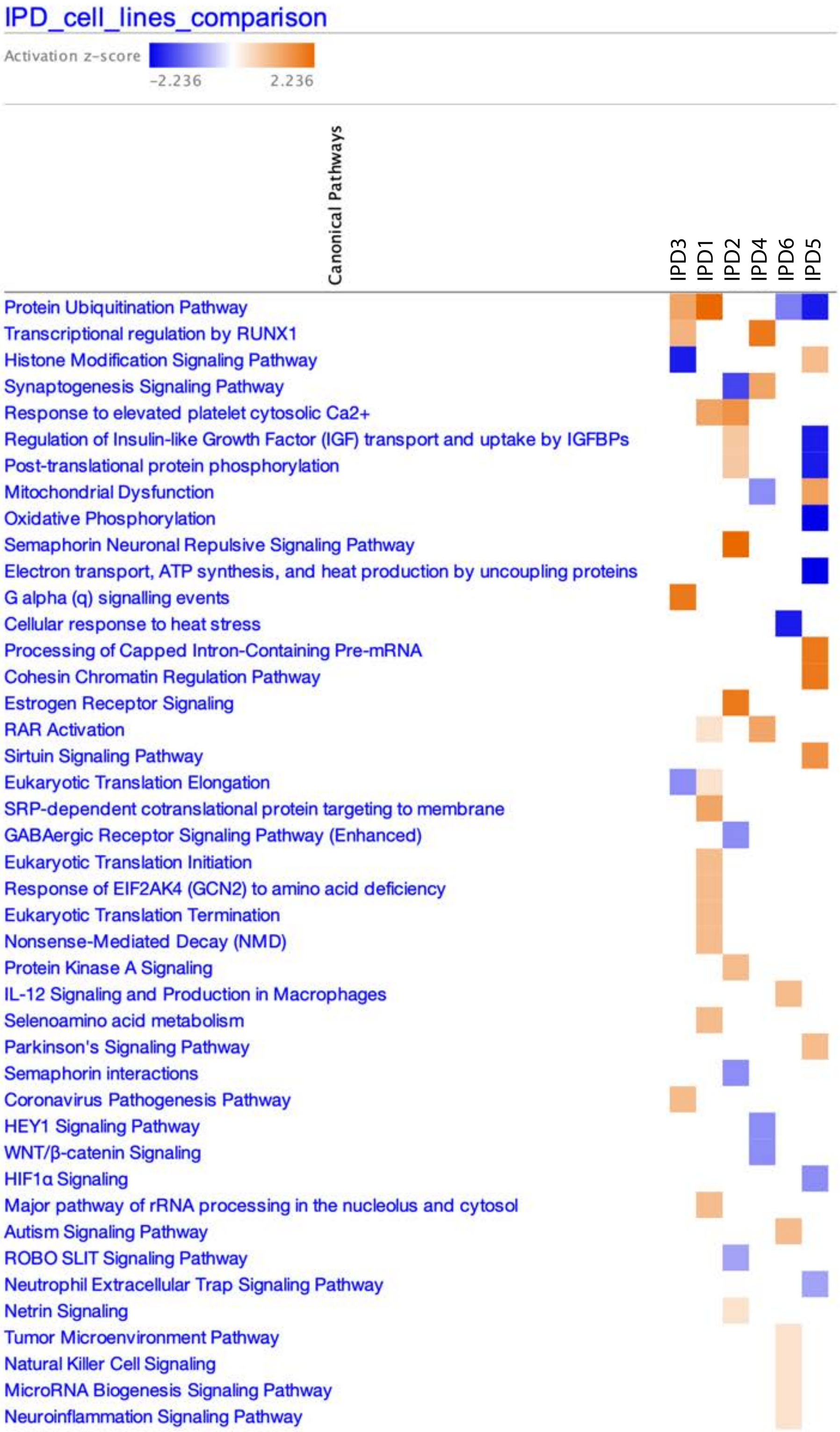

Supplementary Figure 8. Ingenuity pathway analysis to predict the activation level of metabolic and signaling pathways based on log2 fold change (IPD vs CTRL) of the top 100 significant differential expressed genes determined for each IPD lines against all six CTRL samples.

Supplementary Table 1. PD-KG composition.

**Node type**	**Count**	**Comment**
CoreProtein	13	Core proteins are provided in the imaging data
Genes	440	Genes are provided in the transcriptomics data
Cell lines	30	The cell lines for each experiment
CellLineTimePoints	127	Imaging measurement were done at several times points for each cell line.
Feature	1	Currently, there is only the Nuc feature in the integrated datasets. However, the graph model was done to accept more general data structure, with potentialadditional features too.
Normalised Feature	13	Every core proteins is normalised by the features and is specific to cell line time point measurements.
Disease	575	The molecular features (core proteins, genes) being associated with the disease set.
Pathway	670	Pathway (from Reactome) involving the set of core proteins and genes
Drug	445	Drugs targeting the set of molecular features (core proteins, genes)
NeighbourgProtein	1296	Proteins (from IntAct) associated by the protein-protein interactions to the set of molecular features (core proteins and genes)
**Relationship type**	**Count**	**Comment**
ASSOCIATION	908	Relationships of protein-protein association - a subtype of the protein-protein interactions (PPIs)
COLOCALIZATION	4	Protein-protein colocalization relationships
FEATURE_IS_PART_OF	13	Semantic relationships between the Feature and the CellLineTimePoint node types
GBA	291	Relationships indicating the provenience of the transcriptomics from the GBA experiment
GBA_IS_NORMALISED_BY	156	Relationships indicating the provenience of the core proteins from the GBA experiment
GENE_SHARED_DISEASES	3	Network-based computed relationships for genes sharing the same set of diseases

GENE_SHARED_DRUGS	338	Network-based computed relationships for genes being targeted by the same set of drugs
GENE_SHARED_PATHWAYS	843	Network-based computed relationships for genes being involved in the same set of pathways
IPD	576	Relationships indicating the provenience of the transcriptomics from the IPD experiment
IPD_IS_NORMALISED_BY	253	Relationships indicating the provenience of the core proteins from the IPD experiment
IS_ASSOCIATED_WITH	1256	Relationships between the disease and molecular features (core proteins, genes) association
IS_MEASURED_AT	127	Semantic relationships between the CellLines and CellLineTimePoint nodes to incorporate the time point information
IS_PART_OF	13	Semantic relationships conecting the CoreProtein and NormalisedFeature nodes
IS_TARGETED_BY	849	The molecular features (core proteins, genes) - drug targeting relationships; molecular features being core proteins and genes
LRRK2	500	Relationships indicating the provenience of the transcriptomics from the LRRK2 experiment
LRRK2_IS_NORMALISED_BY	42	Relationships indicating the provenience of the core proteins from the LRRK2 experiment
MIRO1	194	Relationships indicating the provenience of the transcriptomics from the MIRO1 experiment
MIRO1_IS_NORMALISED_BY	125	Relationships indicating the provenience of the core proteins from the MIRO1 experiment
PARTICIPATES_IN	1406	Relationships of pathway involment for meolecular features (core proteins, genes)
PHYSICAL_ASSOCIATION	16	Relationships of protein-protein physical association - a subtype of the protein-protein interactions (PPIs)
PROTEIN_GENE_SHARED_PATHWAYS	46	Network-based computed relationships for core proteins and genes being involved in the same set of pathways
PROXIMITY	186	Relationships of protein-protein proximity - a subtype of the protein-protein interactions (PPIs)

SHARED_DISEASES	64	Network-based computed relationships for core proteins sharing the same set of diseases
SHARED_DRUGS	5	Network-based computed relationships for core proteins being targeted by the same set of drugs
SHARED_PATHWAYS	3	Network-based computed relationships for core proteins being involved in the same set of pathways
SHARED_PROTEINS	7	Network-based computed relationships for core proteins having the same set of protein neigtbours, (defined by on the PPI relationships)
SNCA	190	Relationships indicating the provenience of the transcriptomics from the SNCA experiment
SNCA_IS_NORMALISED_BY	98	Relationships indicating the provenience of the core proteins from the SNCA experiment

Supplementary Table 2. Connectivity of the nodes indicating connectivity between genes from the transcriptomics datasets with other node types in the PD-KG knowledge graph [ref. date February 2025]. For each gene node (given by unique UniProt ids) from the transcriptomics set, we calculated the numbers of connected nodes from each category (including pathways, drugs, diseases, interacting proteins, involving cell lines) as well as the overall number of connected elements. Then, we computed the normalised values specifically to each group by division with the max. number from each group. Thus, the normalised values are in the [0,1] range.

GeneSymbol	GeneUniprotId	ConnectedPathways	NormalisedPathwayScore	ConnectDrugs	NormalisedDrugsScore	ConnectedDiseases	NormalisedDiseasesScore	ConnectedCoreProteins	NormalisedCoreProteinsScore	ConnectedNeighbourProteins	NormalisedNeighbourProteinsScore	ConnectedCellLineTimePoints	NormalisedCellLineTimePointsScore	ConnectedNodes	NormalisedNodesScore
RPS27A	P62979	205	1.2000	1	0.0161	0	0.0000	4	1.2000	0	0.0000	5	0.3571	215	1.2000
GFAP	P14136	2	0.0098	27	0.4355	177	1.2000	2	0.5000	1	0.5000	2	0.1429	211	0.9814
TH	P07101	1	0.0049	16	0.2581	125	0.7062	1	0.2500	0	0.0000	3	0.2143	146	0.6791
TUBA1A	Q71U36	37	0.1805	25	0.4032	0	0.0000	2	0.5000	0	0.0000	7	0.5000	71	0.3302
GABRG3	Q99928	2	0.0098	62	1.2000	0	0.0000	0	0.0000	0	0.0000	2	0.1429	66	0.3070
TUBB2A	Q13885	31	0.1512	26	0.4194	0	0.0000	2	0.5000	0	0.0000	6	0.4286	65	0.3023
PSMD5	Q16401	59	0.2878	2	0.0323	0	0.0000	1	0.2500	0	0.0000	2	0.1429	64	0.2977
TUBB2B	Q9BVA1	31	0.1512	24	0.3871	0	0.0000	1	0.2500	0	0.0000	7	0.5000	63	0.2930
TUBA1B	P68363	31	0.1512	24	0.3871	0	0.0000	1	0.2500	0	0.0000	5	0.3571	61	0.2837
MAP2	P11137	0	0.0000	2	0.0323	39	0.2203	0	0.0000	2	1.2000	5	0.3571	48	0.2233
CDKN1A	P38936	25	0.1220	19	0.3065	0	0.0000	2	0.5000	0	0.0000	2	0.1429	48	0.2233
ACTB	P60709	34	0.1659	2	0.0323	0	0.0000	1	0.2500	0	0.0000	7	0.5000	44	0.2047
TUBB	P07437	9	0.0439	26	0.4194	0	0.0000	1	0.2500	0	0.0000	2	0.1429	38	0.1767
AGTR1	P30556	4	0.0195	28	0.4516	0	0.0000	0	0.0000	0	0.0000	3	0.2143	35	0.1628
HGF	P14210	18	0.0878	13	0.2097	0	0.0000	1	0.2500	0	0.0000	3	0.2143	35	0.1628
VEGFA	P15692	7	0.0341	25	0.4032	0	0.0000	1	0.2500	0	0.0000	2	0.1429	35	0.1628
ANXA1	P04083	5	0.0244	23	0.3710	0	0.0000	1	0.2500	0	0.0000	6	0.4286	35	0.1628
ACTG1	P63261	26	0.1268	1	0.0161	0	0.0000	1	0.2500	0	0.0000	7	0.5000	35	0.1628
KDR	P35968	6	0.0293	26	0.4194	0	0.0000	0	0.0000	0	0.0000	2	0.1429	34	0.1581

FGFR2	P21802	5	0.0244	25	0.4032	0	0.0000	0	0.0000	0	0.0000	3	0.2143	33	0.1535
GNG2	P59768	28	0.1366	0	0.0000	0	0.0000	0	0.0000	0	0.0000	2	0.1429	30	0.1395
APOA1	P02647	17	0.0829	7	0.1129	0	0.0000	0	0.0000	0	0.0000	6	0.4286	30	0.1395
PRKCA	P17252	20	0.0976	7	0.1129	0	0.0000	0	0.0000	0	0.0000	3	0.2143	30	0.1395
TSPO	P30536	1	0.0049	26	0.4194	0	0.0000	0	0.0000	0	0.0000	2	0.1429	29	0.1349
TLR4	O00206	17	0.0829	9	0.1452	0	0.0000	0	0.0000	0	0.0000	2	0.1429	28	0.1302
TSPO	B1AH88	0	0.0000	26	0.4194	0	0.0000	0	0.0000	0	0.0000	2	0.1429	28	0.1302
GRIA2	P42262	5	0.0244	14	0.2258	0	0.0000	0	0.0000	0	0.0000	8	0.5714	27	0.1256
SMN2	Q16637	2	0.0098	18	0.2903	0	0.0000	0	0.0000	0	0.0000	6	0.4286	26	0.1209
RPS7	P62081	19	0.0927	1	0.0161	0	0.0000	0	0.0000	0	0.0000	6	0.4286	26	0.1209
HTR3A	P46098	1	0.0049	22	0.3548	0	0.0000	0	0.0000	0	0.0000	2	0.1429	25	0.1163
RPS4X	P62701	18	0.0878	1	0.0161	0	0.0000	0	0.0000	0	0.0000	6	0.4286	25	0.1163
RPS24	P62847	18	0.0878	1	0.0161	0	0.0000	0	0.0000	0	0.0000	5	0.3571	24	0.1116
GNAS	P63092	12	0.0585	6	0.0968	0	0.0000	1	0.2500	0	0.0000	5	0.3571	24	0.1116
GNAS	Q5JWF2	12	0.0585	6	0.0968	0	0.0000	1	0.2500	0	0.0000	5	0.3571	24	0.1116
RPL13	P26373	13	0.0634	3	0.0484	0	0.0000	0	0.0000	0	0.0000	5	0.3571	21	0.0977
PRKCB	P05771	10	0.0488	5	0.0806	0	0.0000	0	0.0000	0	0.0000	6	0.4286	21	0.0977
RPL27A	P46776	14	0.0683	1	0.0161	0	0.0000	0	0.0000	0	0.0000	5	0.3571	20	0.0930
RPL18A	Q02543	13	0.0634	1	0.0161	0	0.0000	0	0.0000	0	0.0000	6	0.4286	20	0.0930
SHH	Q15465	10	0.0488	3	0.0484	0	0.0000	1	0.2500	0	0.0000	6	0.4286	20	0.0930
RPL6	Q02878	13	0.0634	1	0.0161	0	0.0000	0	0.0000	0	0.0000	6	0.4286	20	0.0930
TFRC	P02786	18	0.0878	0	0.0000	0	0.0000	0	0.0000	0	0.0000	2	0.1429	20	0.0930
ADCY1	Q08828	14	0.0683	2	0.0323	0	0.0000	1	0.2500	0	0.0000	3	0.2143	20	0.0930
DCN	P07585	12	0.0585	2	0.0323	0	0.0000	0	0.0000	0	0.0000	6	0.4286	20	0.0930
SLC2A1	P11166	5	0.0244	12	0.1935	0	0.0000	0	0.0000	0	0.0000	2	0.1429	19	0.0884
RPL37A	P61513	13	0.0634	1	0.0161	0	0.0000	0	0.0000	0	0.0000	5	0.3571	19	0.0884
RPL38	P63173	13	0.0634	1	0.0161	0	0.0000	0	0.0000	0	0.0000	5	0.3571	19	0.0884

SLC12A1	Q13621	2	0.0098	13	0.2097	0	0.0000	0	0.0000	0	0.0000	3	0.2143	18	0.0837
CD44	P16070	6	0.0293	6	0.0968	0	0.0000	0	0.0000	0	0.0000	6	0.4286	18	0.0837
SYT1	P21579	11	0.0537	1	0.0161	0	0.0000	0	0.0000	0	0.0000	5	0.3571	17	0.0791
HES1	Q14469	9	0.0439	1	0.0161	0	0.0000	0	0.0000	0	0.0000	6	0.4286	16	0.0744
VIM	P08670	7	0.0341	1	0.0161	0	0.0000	3	0.7500	0	0.0000	5	0.3571	16	0.0744
CBX4	O00257	8	0.0390	0	0.0000	0	0.0000	1	0.2500	0	0.0000	6	0.4286	15	0.0698
ATP1A2	P50993	3	0.0146	6	0.0968	0	0.0000	0	0.0000	0	0.0000	6	0.4286	15	0.0698
P4HB	P07237	11	0.0537	2	0.0323	0	0.0000	0	0.0000	0	0.0000	2	0.1429	15	0.0698
SST	P61278	3	0.0146	9	0.1452	0	0.0000	0	0.0000	0	0.0000	2	0.1429	14	0.0651
DSP	P15924	6	0.0293	1	0.0161	0	0.0000	1	0.2500	0	0.0000	6	0.4286	14	0.0651
MAB21L1	Q13394	0	0.0000	0	0.0000	0	0.0000	0	0.0000	0	0.0000	14	1.2000	14	0.0651
PTPRD	P23468	2	0.0098	5	0.0806	0	0.0000	0	0.0000	0	0.0000	7	0.5000	14	0.0651
MORF4L2	Q15014	1	0.0049	0	0.0000	0	0.0000	0	0.0000	0	0.0000	13	0.9286	14	0.0651
CHAT	P28329	2	0.0098	8	0.1290	0	0.0000	0	0.0000	0	0.0000	3	0.2143	13	0.0605
ROBO2	Q9HCK4	6	0.0293	0	0.0000	0	0.0000	0	0.0000	0	0.0000	7	0.5000	13	0.0605
SPON1	Q9HCB6	2	0.0098	0	0.0000	0	0.0000	0	0.0000	0	0.0000	11	0.7857	13	0.0605
FDPS	P14324	2	0.0098	9	0.1452	0	0.0000	0	0.0000	0	0.0000	2	0.1429	13	0.0605
VEGFB	P49765	3	0.0146	4	0.0645	0	0.0000	0	0.0000	0	0.0000	6	0.4286	13	0.0605
LUM	P51884	7	0.0341	0	0.0000	0	0.0000	0	0.0000	0	0.0000	6	0.4286	13	0.0605
TIMP1	P01033	6	0.0293	0	0.0000	0	0.0000	1	0.2500	0	0.0000	6	0.4286	13	0.0605
DSG2	Q14126	6	0.0293	0	0.0000	0	0.0000	1	0.2500	0	0.0000	6	0.4286	13	0.0605
DPP6	P42658	0	0.0000	2	0.0323	0	0.0000	0	0.0000	0	0.0000	10	0.7143	12	0.0558
MEIS2	O14770	0	0.0000	0	0.0000	0	0.0000	1	0.2500	0	0.0000	11	0.7857	12	0.0558
RXRG	P48443	2	0.0098	7	0.1129	0	0.0000	0	0.0000	0	0.0000	3	0.2143	12	0.0558
MYL6	P60660	7	0.0341	0	0.0000	0	0.0000	0	0.0000	0	0.0000	5	0.3571	12	0.0558
DNER	Q8NFT8	1	0.0049	0	0.0000	0	0.0000	0	0.0000	0	0.0000	11	0.7857	12	0.0558

PGK1	P00558	3	0.0146	1	0.0161	0	0.0000	0	0.0000	0	0.0000	8	0.5714	12	0.0558
SPARC	P09486	4	0.0195	0	0.0000	0	0.0000	2	0.5000	0	0.0000	6	0.4286	12	0.0558
LDHA	P00338	2	0.0098	0	0.0000	0	0.0000	2	0.5000	0	0.0000	8	0.5714	12	0.0558
APOC3	P02656	4	0.0195	2	0.0323	0	0.0000	0	0.0000	0	0.0000	6	0.4286	12	0.0558
NKX6-1	P78426	2	0.0098	2	0.0323	0	0.0000	2	0.5000	0	0.0000	6	0.4286	12	0.0558
CHCHD2	Q9Y6H1	2	0.0098	0	0.0000	0	0.0000	0	0.0000	0	0.0000	10	0.7143	12	0.0558
CXCR4	P61073	6	0.0293	3	0.0484	0	0.0000	1	0.2500	0	0.0000	2	0.1429	12	0.0558
ENO2	P09104	2	0.0098	8	0.1290	0	0.0000	0	0.0000	0	0.0000	2	0.1429	12	0.0558
PLCB1	Q9NQ66	8	0.0390	1	0.0161	0	0.0000	0	0.0000	0	0.0000	2	0.1429	11	0.0512
GNAS	O95467	0	0.0000	6	0.0968	0	0.0000	0	0.0000	0	0.0000	5	0.3571	11	0.0512
FMO3	P31513	2	0.0098	7	0.1129	0	0.0000	0	0.0000	0	0.0000	2	0.1429	11	0.0512
GAPDH	P04406	2	0.0098	4	0.0645	0	0.0000	0	0.0000	0	0.0000	5	0.3571	11	0.0512
TCEAL7	Q9BRU2	0	0.0000	0	0.0000	0	0.0000	0	0.0000	0	0.0000	11	0.7857	11	0.0512
SHOX2	O60902	0	0.0000	0	0.0000	0	0.0000	0	0.0000	0	0.0000	11	0.7857	11	0.0512
APOA4	P06727	5	0.0244	0	0.0000	0	0.0000	0	0.0000	0	0.0000	6	0.4286	11	0.0512
GNAS	P84996	0	0.0000	6	0.0968	0	0.0000	0	0.0000	0	0.0000	5	0.3571	11	0.0512
FTL	P02792	4	0.0195	0	0.0000	0	0.0000	0	0.0000	0	0.0000	7	0.5000	11	0.0512
ROBO1	Q9Y6N7	9	0.0439	0	0.0000	0	0.0000	0	0.0000	0	0.0000	2	0.1429	11	0.0512
ANXA2	P07355	4	0.0195	1	0.0161	0	0.0000	0	0.0000	0	0.0000	6	0.4286	11	0.0512
SULF1	Q8IWU6	0	0.0000	0	0.0000	0	0.0000	0	0.0000	0	0.0000	11	0.7857	11	0.0512
PSAP	P07602	5	0.0244	0	0.0000	0	0.0000	0	0.0000	0	0.0000	5	0.3571	10	0.0465
EPHA5	P54756	3	0.0146	2	0.0323	0	0.0000	0	0.0000	0	0.0000	5	0.3571	10	0.0465
JUND	P17535	2	0.0098	1	0.0161	0	0.0000	1	0.2500	0	0.0000	6	0.4286	10	0.0465
GAD1	Q99259	3	0.0146	0	0.0000	0	0.0000	0	0.0000	0	0.0000	7	0.5000	10	0.0465
DICER1	Q9UPY3	5	0.0244	0	0.0000	0	0.0000	0	0.0000	0	0.0000	5	0.3571	10	0.0465
ENO1	P06733	3	0.0146	0	0.0000	0	0.0000	0	0.0000	0	0.0000	7	0.5000	10	0.0465
KRT18	P05783	2	0.0098	2	0.0323	0	0.0000	0	0.0000	0	0.0000	6	0.4286	10	0.0465

ARF4	P18085	3	0.0146	0	0.0000	0	0.0000	1	0.2500	0	0.0000	6	0.4286	10	0.0465
NPY	P01303	3	0.0146	5	0.0806	0	0.0000	0	0.0000	0	0.0000	2	0.1429	10	0.0465
RBP1	P09455	3	0.0146	0	0.0000	0	0.0000	1	0.2500	0	0.0000	6	0.4286	10	0.0465
CD9	P21926	3	0.0146	0	0.0000	0	0.0000	0	0.0000	0	0.0000	6	0.4286	9	0.0419
RBFOX1	Q9NWB 1	1	0.0049	0	0.0000	0	0.0000	0	0.0000	0	0.0000	8	0.5714	9	0.0419
WNT4	P56705	6	0.0293	0	0.0000	0	0.0000	0	0.0000	0	0.0000	3	0.2143	9	0.0419
TFF3	Q07654	1	0.0049	2	0.0323	0	0.0000	0	0.0000	0	0.0000	6	0.4286	9	0.0419
FTH1	P02794	4	0.0195	0	0.0000	0	0.0000	0	0.0000	0	0.0000	5	0.3571	9	0.0419
CPLX1	O14810	6	0.0293	0	0.0000	0	0.0000	0	0.0000	0	0.0000	3	0.2143	9	0.0419
L1CAM	P32004	5	0.0244	0	0.0000	0	0.0000	1	0.2500	0	0.0000	3	0.2143	9	0.0419
MAP1LC 3A	Q9H492	3	0.0146	0	0.0000	0	0.0000	0	0.0000	0	0.0000	6	0.4286	9	0.0419
CSRP1	P21291	1	0.0049	0	0.0000	0	0.0000	0	0.0000	0	0.0000	8	0.5714	9	0.0419
PKM	P14618	1	0.0049	6	0.0968	0	0.0000	0	0.0000	0	0.0000	2	0.1429	9	0.0419
DDIT4	Q9NX09	1	0.0049	6	0.0968	0	0.0000	0	0.0000	0	0.0000	2	0.1429	9	0.0419
ZNF558	Q96NG5	1	0.0049	0	0.0000	0	0.0000	0	0.0000	0	0.0000	8	0.5714	9	0.0419
KCND2	Q9NZV8	2	0.0098	4	0.0645	0	0.0000	0	0.0000	0	0.0000	3	0.2143	9	0.0419
FOXA1	P55317	2	0.0098	0	0.0000	0	0.0000	1	0.2500	0	0.0000	6	0.4286	9	0.0419
ANK3	Q12955	2	0.0098	0	0.0000	0	0.0000	1	0.2500	0	0.0000	5	0.3571	8	0.0372
KRT8	P05787	2	0.0098	0	0.0000	0	0.0000	0	0.0000	0	0.0000	6	0.4286	8	0.0372
WNT7B	P56706	2	0.0098	0	0.0000	0	0.0000	0	0.0000	0	0.0000	6	0.4286	8	0.0372
SLC17A6	Q9P2U8	1	0.0049	0	0.0000	0	0.0000	0	0.0000	0	0.0000	7	0.5000	8	0.0372
MAB21L 2	Q9Y586	0	0.0000	0	0.0000	0	0.0000	0	0.0000	0	0.0000	8	0.5714	8	0.0372
POU3F1	Q03052	3	0.0146	0	0.0000	0	0.0000	2	0.5000	0	0.0000	3	0.2143	8	0.0372
CD81	P60033	2	0.0098	0	0.0000	0	0.0000	0	0.0000	0	0.0000	6	0.4286	8	0.0372
UTS2	O95399	2	0.0098	0	0.0000	0	0.0000	0	0.0000	0	0.0000	6	0.4286	8	0.0372
SLC2A3	P11169	4	0.0195	2	0.0323	0	0.0000	0	0.0000	0	0.0000	2	0.1429	8	0.0372

LBX1	P52954	0	0.0000	0	0.0000	0	0.0000	0	0.0000	0	0.0000	8	0.5714	8	0.0372
NR2F2	P24468	1	0.0049	0	0.0000	0	0.0000	1	0.2500	0	0.0000	6	0.4286	8	0.0372
SRP14	P37108	2	0.0098	0	0.0000	0	0.0000	0	0.0000	0	0.0000	6	0.4286	8	0.0372
CHL1	O00533	1	0.0049	0	0.0000	0	0.0000	0	0.0000	0	0.0000	7	0.5000	8	0.0372
IGHA1	P01876	2	0.0098	0	0.0000	0	0.0000	0	0.0000	0	0.0000	6	0.4286	8	0.0372
NNAT	Q16517	0	0.0000	0	0.0000	0	0.0000	0	0.0000	0	0.0000	8	0.5714	8	0.0372
TWIST1	Q15672	4	0.0195	1	0.0161	0	0.0000	1	0.2500	0	0.0000	2	0.1429	8	0.0372
IGFBP2	P18065	1	0.0049	0	0.0000	0	0.0000	0	0.0000	0	0.0000	7	0.5000	8	0.0372
CDKN1C	P49918	2	0.0098	0	0.0000	0	0.0000	0	0.0000	0	0.0000	6	0.4286	8	0.0372
GATA2	P23769	3	0.0146	3	0.0484	0	0.0000	0	0.0000	0	0.0000	2	0.1429	8	0.0372
PAX5	Q02548	1	0.0049	0	0.0000	0	0.0000	0	0.0000	0	0.0000	7	0.5000	8	0.0372
PRKX	P51817	4	0.0195	1	0.0161	0	0.0000	0	0.0000	0	0.0000	3	0.2143	8	0.0372
KCNC3	Q14003	1	0.0049	4	0.0645	0	0.0000	0	0.0000	0	0.0000	3	0.2143	8	0.0372
PPFIA2	O75334	6	0.0293	0	0.0000	0	0.0000	0	0.0000	0	0.0000	2	0.1429	8	0.0372
PDK1	Q15118	3	0.0146	3	0.0484	0	0.0000	0	0.0000	0	0.0000	2	0.1429	8	0.0372
EPHA7	Q15375	3	0.0146	2	0.0323	0	0.0000	0	0.0000	0	0.0000	3	0.2143	8	0.0372
PCDHB5	Q9Y5E4	0	0.0000	0	0.0000	0	0.0000	0	0.0000	0	0.0000	8	0.5714	8	0.0372
S100A10	P60903	1	0.0049	1	0.0161	0	0.0000	0	0.0000	0	0.0000	6	0.4286	8	0.0372
TLX1	P31314	0	0.0000	0	0.0000	0	0.0000	0	0.0000	0	0.0000	8	0.5714	8	0.0372
SPARCL 1	Q14515	2	0.0098	0	0.0000	0	0.0000	0	0.0000	0	0.0000	6	0.4286	8	0.0372
FMOD	Q06828	6	0.0293	0	0.0000	0	0.0000	0	0.0000	0	0.0000	2	0.1429	8	0.0372
IRX2	Q9BZI1	1	0.0049	0	0.0000	0	0.0000	0	0.0000	0	0.0000	6	0.4286	7	0.0326
MEIS1	O00470	1	0.0049	0	0.0000	0	0.0000	1	0.2500	0	0.0000	5	0.3571	7	0.0326
TGFB3	P10600	4	0.0195	1	0.0161	0	0.0000	0	0.0000	0	0.0000	2	0.1429	7	0.0326
TIMP2	P16035	2	0.0098	0	0.0000	0	0.0000	0	0.0000	0	0.0000	5	0.3571	7	0.0326
ALCAM	Q13740	1	0.0049	1	0.0161	0	0.0000	0	0.0000	0	0.0000	5	0.3571	7	0.0326
EMP1	P54849	0	0.0000	1	0.0161	0	0.0000	0	0.0000	0	0.0000	6	0.4286	7	0.0326

OTP	Q5XKR4	0	0.0000	0	0.0000	0	0.0000	0	0.0000	0	0.0000	7	0.5000	7	0.0326
S100A6	P06703	0	0.0000	0	0.0000	0	0.0000	1	0.2500	0	0.0000	6	0.4286	7	0.0326
AKAP12	Q02952	2	0.0098	0	0.0000	0	0.0000	0	0.0000	0	0.0000	5	0.3571	7	0.0326
TFPI2	P48307	0	0.0000	1	0.0161	0	0.0000	0	0.0000	0	0.0000	6	0.4286	7	0.0326
IFITM3	Q01628	1	0.0049	0	0.0000	0	0.0000	0	0.0000	0	0.0000	6	0.4286	7	0.0326
IGFBP5	P24593	2	0.0098	0	0.0000	0	0.0000	0	0.0000	0	0.0000	5	0.3571	7	0.0326
LHX4	Q969G2	1	0.0049	0	0.0000	0	0.0000	0	0.0000	0	0.0000	6	0.4286	7	0.0326
EFEMP1	Q12805	1	0.0049	0	0.0000	0	0.0000	0	0.0000	0	0.0000	6	0.4286	7	0.0326
CADM1	Q9BY67	2	0.0098	0	0.0000	0	0.0000	0	0.0000	0	0.0000	5	0.3571	7	0.0326
GRK3	P35626	2	0.0098	0	0.0000	0	0.0000	0	0.0000	0	0.0000	5	0.3571	7	0.0326
TAGLN2	P37802	1	0.0049	0	0.0000	0	0.0000	0	0.0000	0	0.0000	6	0.4286	7	0.0326
ID3	Q02535	1	0.0049	0	0.0000	0	0.0000	0	0.0000	0	0.0000	6	0.4286	7	0.0326
EN1	Q05925	0	0.0000	0	0.0000	0	0.0000	0	0.0000	0	0.0000	7	0.5000	7	0.0326
STMN1	P16949	1	0.0049	2	0.0323	0	0.0000	2	0.5000	0	0.0000	2	0.1429	7	0.0326
CRYAB	P02511	1	0.0049	0	0.0000	0	0.0000	0	0.0000	0	0.0000	6	0.4286	7	0.0326
ADM	P35318	2	0.0098	3	0.0484	0	0.0000	0	0.0000	0	0.0000	2	0.1429	7	0.0326
SCG2	P13521	2	0.0098	0	0.0000	0	0.0000	0	0.0000	0	0.0000	5	0.3571	7	0.0326
SGIP1	Q9BQI5	2	0.0098	0	0.0000	0	0.0000	0	0.0000	0	0.0000	5	0.3571	7	0.0326
GPX3	P22352	1	0.0049	0	0.0000	0	0.0000	0	0.0000	0	0.0000	6	0.4286	7	0.0326
LHX3	Q9UBR4	1	0.0049	0	0.0000	0	0.0000	0	0.0000	0	0.0000	6	0.4286	7	0.0326
ZIM2	Q9NZV7	1	0.0049	0	0.0000	0	0.0000	0	0.0000	0	0.0000	6	0.4286	7	0.0326
CHGB	P05060	2	0.0098	0	0.0000	0	0.0000	0	0.0000	0	0.0000	5	0.3571	7	0.0326
EIF5A	P63241	1	0.0049	0	0.0000	0	0.0000	0	0.0000	0	0.0000	6	0.4286	7	0.0326
EEF1A2	Q05639	1	0.0049	0	0.0000	0	0.0000	0	0.0000	0	0.0000	6	0.4286	7	0.0326
SOX14	O95416	0	0.0000	0	0.0000	0	0.0000	1	0.2500	0	0.0000	6	0.4286	7	0.0326
TPH2	Q8IWU9	1	0.0049	4	0.0645	0	0.0000	0	0.0000	0	0.0000	2	0.1429	7	0.0326
PEG10	Q86TG7	0	0.0000	0	0.0000	0	0.0000	0	0.0000	0	0.0000	7	0.5000	7	0.0326

WLS	Q5T9L3	1	0.0049	0	0.0000	0	0.0000	0	0.0000	0	0.0000	6	0.4286	7	0.0326
EMP3	P54852	0	0.0000	0	0.0000	0	0.0000	0	0.0000	0	0.0000	6	0.4286	6	0.0279
MYT1L	Q9UL68	0	0.0000	1	0.0161	0	0.0000	0	0.0000	0	0.0000	5	0.3571	6	0.0279
CCK	P06307	2	0.0098	2	0.0323	0	0.0000	0	0.0000	0	0.0000	2	0.1429	6	0.0279
PHPT1	Q9NRX4	0	0.0000	0	0.0000	0	0.0000	0	0.0000	0	0.0000	6	0.4286	6	0.0279
FDFT1	P37268	3	0.0146	1	0.0161	0	0.0000	0	0.0000	0	0.0000	2	0.1429	6	0.0279
CRTAC1	Q9NQ79	0	0.0000	0	0.0000	0	0.0000	0	0.0000	0	0.0000	6	0.4286	6	0.0279
ZFP82	Q8N141	0	0.0000	0	0.0000	0	0.0000	0	0.0000	0	0.0000	6	0.4286	6	0.0279
SERF2	P84101	0	0.0000	0	0.0000	0	0.0000	0	0.0000	0	0.0000	6	0.4286	6	0.0279
BEX5	Q5H9J7	0	0.0000	0	0.0000	0	0.0000	0	0.0000	0	0.0000	6	0.4286	6	0.0279
HOXB4	P17483	2	0.0098	0	0.0000	0	0.0000	1	0.2500	0	0.0000	3	0.2143	6	0.0279
TMSB4X	P62328	1	0.0049	0	0.0000	0	0.0000	0	0.0000	0	0.0000	5	0.3571	6	0.0279
MTF2	Q9Y483	1	0.0049	0	0.0000	0	0.0000	0	0.0000	0	0.0000	5	0.3571	6	0.0279
NKX6-2	Q9C056	0	0.0000	0	0.0000	0	0.0000	0	0.0000	0	0.0000	6	0.4286	6	0.0279
SMS	P52788	1	0.0049	0	0.0000	0	0.0000	0	0.0000	0	0.0000	5	0.3571	6	0.0279
IRF6	O14896	2	0.0098	2	0.0323	0	0.0000	0	0.0000	0	0.0000	2	0.1429	6	0.0279
TMSB10	P63313	0	0.0000	1	0.0161	0	0.0000	0	0.0000	0	0.0000	5	0.3571	6	0.0279
TAC3	Q9UHF0	2	0.0098	1	0.0161	0	0.0000	0	0.0000	0	0.0000	3	0.2143	6	0.0279
MAP3K13	O43283	0	0.0000	0	0.0000	0	0.0000	0	0.0000	0	0.0000	6	0.4286	6	0.0279
CPE	P16870	1	0.0049	0	0.0000	0	0.0000	0	0.0000	0	0.0000	5	0.3571	6	0.0279
TCIM	Q9NR00	0	0.0000	0	0.0000	0	0.0000	0	0.0000	0	0.0000	6	0.4286	6	0.0279
NALCN	Q8IZF0	1	0.0049	2	0.0323	0	0.0000	0	0.0000	0	0.0000	3	0.2143	6	0.0279
CHRDL1	Q9BU40	3	0.0146	0	0.0000	0	0.0000	0	0.0000	0	0.0000	3	0.2143	6	0.0279
PGAP1	Q75T13	1	0.0049	0	0.0000	0	0.0000	0	0.0000	0	0.0000	5	0.3571	6	0.0279
SLC8A2	Q9UPR5	3	0.0146	0	0.0000	0	0.0000	0	0.0000	0	0.0000	3	0.2143	6	0.0279
PLK2	Q9NYY3	3	0.0146	0	0.0000	0	0.0000	0	0.0000	0	0.0000	3	0.2143	6	0.0279
BEX3	Q00994	1	0.0049	0	0.0000	0	0.0000	0	0.0000	0	0.0000	5	0.3571	6	0.0279

FGF13	Q92913	1	0.0049	0	0.0000	0	0.0000	0	0.0000	0	0.0000	5	0.3571	6	0.0279
CELF4	Q9BZC1	0	0.0000	1	0.0161	0	0.0000	0	0.0000	0	0.0000	5	0.3571	6	0.0279
MECP2	P51608	0	0.0000	3	0.0484	0	0.0000	1	0.2500	0	0.0000	2	0.1429	6	0.0279
PCLO	Q9Y6V0	1	0.0049	0	0.0000	0	0.0000	0	0.0000	0	0.0000	5	0.3571	6	0.0279
TKTL1	P51854	0	0.0000	0	0.0000	0	0.0000	0	0.0000	0	0.0000	6	0.4286	6	0.0279
PHIP	Q8WWQ0	1	0.0049	0	0.0000	0	0.0000	0	0.0000	0	0.0000	5	0.3571	6	0.0279
ELAVL3	Q14576	0	0.0000	0	0.0000	0	0.0000	0	0.0000	0	0.0000	6	0.4286	6	0.0279
VSX2	P58304	0	0.0000	0	0.0000	0	0.0000	0	0.0000	0	0.0000	6	0.4286	6	0.0279
SRGAP1	Q7Z6B7	4	0.0195	0	0.0000	0	0.0000	0	0.0000	0	0.0000	2	0.1429	6	0.0279
KITLG	P21583	0	0.0000	0	0.0000	0	0.0000	0	0.0000	0	0.0000	6	0.4286	6	0.0279
NR2F1	P10589	1	0.0049	0	0.0000	0	0.0000	0	0.0000	0	0.0000	5	0.3571	6	0.0279
PDE1C	Q14123	1	0.0049	2	0.0323	0	0.0000	0	0.0000	0	0.0000	3	0.2143	6	0.0279
IER5L	Q5T953	0	0.0000	0	0.0000	0	0.0000	0	0.0000	0	0.0000	6	0.4286	6	0.0279
TMEM47	Q9BQJ4	0	0.0000	0	0.0000	0	0.0000	0	0.0000	0	0.0000	6	0.4286	6	0.0279
ZNF608	Q9ULD9	0	0.0000	0	0.0000	0	0.0000	1	0.2500	0	0.0000	5	0.3571	6	0.0279
ZNF638	Q14966	1	0.0049	0	0.0000	0	0.0000	0	0.0000	0	0.0000	5	0.3571	6	0.0279
PCDHB2	Q9Y5E7	0	0.0000	0	0.0000	0	0.0000	0	0.0000	0	0.0000	6	0.4286	6	0.0279
ZNF37A	P17032	1	0.0049	0	0.0000	0	0.0000	0	0.0000	0	0.0000	5	0.3571	6	0.0279
NNMT	P40261	3	0.0146	1	0.0161	0	0.0000	0	0.0000	0	0.0000	2	0.1429	6	0.0279
PCDH7	O60245	3	0.0146	0	0.0000	0	0.0000	0	0.0000	0	0.0000	3	0.2143	6	0.0279
MLXIPL	Q9NP71	4	0.0195	0	0.0000	0	0.0000	0	0.0000	0	0.0000	2	0.1429	6	0.0279
LSAMP	Q13449	1	0.0049	0	0.0000	0	0.0000	0	0.0000	0	0.0000	5	0.3571	6	0.0279
NCS1	P62166	0	0.0000	0	0.0000	0	0.0000	0	0.0000	0	0.0000	6	0.4286	6	0.0279
ATF7IP	Q6VMQ6	1	0.0049	0	0.0000	0	0.0000	0	0.0000	0	0.0000	5	0.3571	6	0.0279
SEMA3A	Q14563	3	0.0146	0	0.0000	0	0.0000	0	0.0000	0	0.0000	3	0.2143	6	0.0279
MTRNR2L1	P0CJ68	0	0.0000	0	0.0000	0	0.0000	0	0.0000	0	0.0000	5	0.3571	5	0.0233

SEC61G	P60059	3	0.0146	0	0.0000	0	0.0000	0	0.0000	0	0.0000	2	0.1429	5	0.0233
MTRNR2L3	P0CJ70	0	0.0000	0	0.0000	0	0.0000	0	0.0000	0	0.0000	5	0.3571	5	0.0233
LEFTY2	O00292	3	0.0146	0	0.0000	0	0.0000	0	0.0000	0	0.0000	2	0.1429	5	0.0233
TCEAL2	Q9H3H9	0	0.0000	0	0.0000	0	0.0000	0	0.0000	0	0.0000	5	0.3571	5	0.0233
PCSK5	Q92824	2	0.0098	0	0.0000	0	0.0000	0	0.0000	0	0.0000	3	0.2143	5	0.0233
TMEM132B	Q14DG7	0	0.0000	0	0.0000	0	0.0000	0	0.0000	0	0.0000	5	0.3571	5	0.0233
SLC16A3	O15427	2	0.0098	1	0.0161	0	0.0000	0	0.0000	0	0.0000	2	0.1429	5	0.0233
RUNX1T1	Q06455	0	0.0000	0	0.0000	0	0.0000	0	0.0000	0	0.0000	5	0.3571	5	0.0233
PMFBP1	Q8TBY8	0	0.0000	0	0.0000	0	0.0000	0	0.0000	0	0.0000	5	0.3571	5	0.0233
N4BP2	Q86UW6	0	0.0000	0	0.0000	0	0.0000	0	0.0000	0	0.0000	5	0.3571	5	0.0233
CLSTN2	Q9H4D0	0	0.0000	0	0.0000	0	0.0000	0	0.0000	0	0.0000	5	0.3571	5	0.0233
MAGED1	Q9Y5V3	2	0.0098	0	0.0000	0	0.0000	1	0.2500	0	0.0000	2	0.1429	5	0.0233
NOVA1	P51513	0	0.0000	0	0.0000	0	0.0000	0	0.0000	0	0.0000	5	0.3571	5	0.0233
VSNL1	P62760	0	0.0000	0	0.0000	0	0.0000	0	0.0000	0	0.0000	5	0.3571	5	0.0233
AMY2A	P04746	1	0.0049	1	0.0161	0	0.0000	0	0.0000	0	0.0000	3	0.2143	5	0.0233
ADARB2	Q9NS39	0	0.0000	0	0.0000	0	0.0000	0	0.0000	0	0.0000	5	0.3571	5	0.0233
CCNI	Q14094	0	0.0000	0	0.0000	0	0.0000	0	0.0000	0	0.0000	5	0.3571	5	0.0233
CNTNAP2	Q9UHC6	0	0.0000	0	0.0000	0	0.0000	0	0.0000	0	0.0000	5	0.3571	5	0.0233
PCDH17	O14917	0	0.0000	0	0.0000	0	0.0000	0	0.0000	0	0.0000	5	0.3571	5	0.0233
ELAVL2	Q12926	0	0.0000	0	0.0000	0	0.0000	0	0.0000	0	0.0000	5	0.3571	5	0.0233
SYT4	Q9H2B2	0	0.0000	0	0.0000	0	0.0000	0	0.0000	0	0.0000	5	0.3571	5	0.0233
ESRRG	P62508	1	0.0049	1	0.0161	0	0.0000	0	0.0000	0	0.0000	3	0.2143	5	0.0233
MAGI3	Q5TCQ9	0	0.0000	0	0.0000	0	0.0000	0	0.0000	0	0.0000	5	0.3571	5	0.0233
GPM6B	Q13491	0	0.0000	0	0.0000	0	0.0000	0	0.0000	0	0.0000	5	0.3571	5	0.0233
RGS7	P49802	2	0.0098	0	0.0000	0	0.0000	0	0.0000	0	0.0000	3	0.2143	5	0.0233

SLITRK3	O94933	2	0.0098	0	0.0000	0	0.0000	0	0.0000	0	0.0000	3	0.2143	5	0.0233
HMGCS1	Q01581	3	0.0146	0	0.0000	0	0.0000	0	0.0000	0	0.0000	2	0.1429	5	0.0233
HAP1	P54257	0	0.0000	0	0.0000	0	0.0000	0	0.0000	0	0.0000	5	0.3571	5	0.0233
BEX2	Q9BXY8	0	0.0000	0	0.0000	0	0.0000	0	0.0000	0	0.0000	5	0.3571	5	0.0233
DUSP9	Q99956	3	0.0146	0	0.0000	0	0.0000	0	0.0000	0	0.0000	2	0.1429	5	0.0233
DNAJC12	Q9UKB3	0	0.0000	0	0.0000	0	0.0000	0	0.0000	0	0.0000	5	0.3571	5	0.0233
VASH2	Q86V25	1	0.0049	0	0.0000	0	0.0000	1	0.2500	0	0.0000	3	0.2143	5	0.0233
ONECUT2	O95948	0	0.0000	0	0.0000	0	0.0000	0	0.0000	0	0.0000	5	0.3571	5	0.0233
PDZRN4	Q6ZMN7	0	0.0000	0	0.0000	0	0.0000	0	0.0000	0	0.0000	5	0.3571	5	0.0233
RFX4	Q33E94	0	0.0000	0	0.0000	0	0.0000	0	0.0000	0	0.0000	5	0.3571	5	0.0233
FHL1	Q13642	0	0.0000	0	0.0000	0	0.0000	0	0.0000	0	0.0000	5	0.3571	5	0.0233
PHF14	O94880	0	0.0000	0	0.0000	0	0.0000	0	0.0000	0	0.0000	5	0.3571	5	0.0233
TCEAL4	Q96EI5	0	0.0000	0	0.0000	0	0.0000	0	0.0000	0	0.0000	5	0.3571	5	0.0233
UGT2B4	P06133	2	0.0098	1	0.0161	0	0.0000	0	0.0000	0	0.0000	2	0.1429	5	0.0233
PCDH9	Q9HC56	0	0.0000	0	0.0000	0	0.0000	0	0.0000	0	0.0000	5	0.3571	5	0.0233
TBX2	Q13207	1	0.0049	1	0.0161	0	0.0000	0	0.0000	0	0.0000	3	0.2143	5	0.0233
EGLN3	Q9H6Z9	1	0.0049	2	0.0323	0	0.0000	0	0.0000	0	0.0000	2	0.1429	5	0.0233
OR2L13	Q8N349	2	0.0098	0	0.0000	0	0.0000	0	0.0000	0	0.0000	2	0.1429	4	0.0186
PGM2L1	Q6PCE3	1	0.0049	0	0.0000	0	0.0000	0	0.0000	0	0.0000	3	0.2143	4	0.0186
TPI1	P60174	2	0.0098	0	0.0000	0	0.0000	0	0.0000	0	0.0000	2	0.1429	4	0.0186
AMY1B	P0DTE7	1	0.0049	0	0.0000	0	0.0000	0	0.0000	0	0.0000	3	0.2143	4	0.0186
AMY1C	P0DTE8	1	0.0049	0	0.0000	0	0.0000	0	0.0000	0	0.0000	3	0.2143	4	0.0186
KCNIP1	Q9NZI2	1	0.0049	1	0.0161	0	0.0000	0	0.0000	0	0.0000	2	0.1429	4	0.0186
AMY2B	P19961	1	0.0049	0	0.0000	0	0.0000	0	0.0000	0	0.0000	3	0.2143	4	0.0186
CLVS2	Q5SYC1	1	0.0049	0	0.0000	0	0.0000	0	0.0000	0	0.0000	3	0.2143	4	0.0186
COLEC12	Q5KU26	2	0.0098	0	0.0000	0	0.0000	0	0.0000	0	0.0000	2	0.1429	4	0.0186

USP11	P51784	2	0.0098	0	0.0000	0	0.0000	0	0.0000	0	0.0000	2	0.1429	4	0.0186
GOLIM4	O00461	1	0.0049	0	0.0000	0	0.0000	0	0.0000	0	0.0000	3	0.2143	4	0.0186
LHX2	P50458	2	0.0098	0	0.0000	0	0.0000	0	0.0000	0	0.0000	2	0.1429	4	0.0186
KLHL13	Q9P2N7	2	0.0098	0	0.0000	0	0.0000	0	0.0000	0	0.0000	2	0.1429	4	0.0186
ADAM22	Q9P0K1	1	0.0049	0	0.0000	0	0.0000	0	0.0000	0	0.0000	3	0.2143	4	0.0186
RNPC3	Q96LT9	1	0.0049	0	0.0000	0	0.0000	0	0.0000	0	0.0000	3	0.2143	4	0.0186
HOXB9	P17482	0	0.0000	0	0.0000	0	0.0000	1	0.2500	0	0.0000	3	0.2143	4	0.0186
CADM3	Q8N126	2	0.0098	0	0.0000	0	0.0000	0	0.0000	0	0.0000	2	0.1429	4	0.0186
ETV1	P50549	0	0.0000	1	0.0161	0	0.0000	0	0.0000	0	0.0000	3	0.2143	4	0.0186
AMY1A	P0DUB6	1	0.0049	0	0.0000	0	0.0000	0	0.0000	0	0.0000	3	0.2143	4	0.0186
SHANK1	Q9Y566	1	0.0049	0	0.0000	0	0.0000	0	0.0000	0	0.0000	3	0.2143	4	0.0186
TBX20	Q9UMR3	1	0.0049	0	0.0000	0	0.0000	0	0.0000	0	0.0000	3	0.2143	4	0.0186
ASCL1	P50553	1	0.0049	0	0.0000	0	0.0000	0	0.0000	0	0.0000	3	0.2143	4	0.0186
TMEFF2	Q9UIK5	0	0.0000	1	0.0161	0	0.0000	0	0.0000	0	0.0000	3	0.2143	4	0.0186
CTSF	Q9UBX1	1	0.0049	0	0.0000	0	0.0000	1	0.2500	0	0.0000	2	0.1429	4	0.0186
BCL11A	Q9H165	2	0.0098	0	0.0000	0	0.0000	0	0.0000	0	0.0000	2	0.1429	4	0.0186
INPP5F	Q9Y2H2	1	0.0049	0	0.0000	0	0.0000	0	0.0000	0	0.0000	3	0.2143	4	0.0186
CBLN1	P23435	0	0.0000	0	0.0000	0	0.0000	0	0.0000	0	0.0000	4	0.2857	4	0.0186
BRS3	P32247	2	0.0098	0	0.0000	0	0.0000	0	0.0000	0	0.0000	2	0.1429	4	0.0186
PLTP	P55058	0	0.0000	2	0.0323	0	0.0000	0	0.0000	0	0.0000	2	0.1429	4	0.0186
PRDX4	Q13162	1	0.0049	1	0.0161	0	0.0000	0	0.0000	0	0.0000	2	0.1429	4	0.0186
FRAS1	Q86XX4	0	0.0000	0	0.0000	0	0.0000	1	0.2500	0	0.0000	3	0.2143	4	0.0186
TRDN	Q13061	2	0.0098	0	0.0000	0	0.0000	0	0.0000	0	0.0000	2	0.1429	4	0.0186
ATP6AP 2	O75787	2	0.0098	0	0.0000	0	0.0000	0	0.0000	0	0.0000	2	0.1429	4	0.0186
MAP1B	P46821	1	0.0049	0	0.0000	0	0.0000	1	0.2500	0	0.0000	2	0.1429	4	0.0186
OTOF	Q9HC10	2	0.0098	0	0.0000	0	0.0000	0	0.0000	0	0.0000	2	0.1429	4	0.0186
EBF1	Q9UH73	2	0.0098	0	0.0000	0	0.0000	0	0.0000	0	0.0000	2	0.1429	4	0.0186

KHDRBS3	O75525	1	0.0049	0	0.0000	0	0.0000	0	0.0000	0	0.0000	3	0.2143	4	0.0186
CNTN4	Q8IWV2	1	0.0049	1	0.0161	0	0.0000	0	0.0000	0	0.0000	2	0.1429	4	0.0186
SLC32A1	Q9H598	2	0.0098	0	0.0000	0	0.0000	0	0.0000	0	0.0000	2	0.1429	4	0.0186
GPD1	P21695	1	0.0049	0	0.0000	0	0.0000	0	0.0000	0	0.0000	3	0.2143	4	0.0186
PAPPA	Q13219	1	0.0049	0	0.0000	0	0.0000	0	0.0000	0	0.0000	3	0.2143	4	0.0186
FOXP1	Q9H334	0	0.0000	0	0.0000	0	0.0000	1	0.2500	0	0.0000	3	0.2143	4	0.0186
CHST9	Q7L1S5	0	0.0000	0	0.0000	0	0.0000	0	0.0000	0	0.0000	3	0.2143	3	0.0140
UCHL1	P09936	1	0.0049	0	0.0000	0	0.0000	0	0.0000	0	0.0000	2	0.1429	3	0.0140
NLGN4Y	Q8NFZ3	1	0.0049	0	0.0000	0	0.0000	0	0.0000	0	0.0000	2	0.1429	3	0.0140
STXBP6	Q8NFX7	0	0.0000	0	0.0000	0	0.0000	0	0.0000	0	0.0000	3	0.2143	3	0.0140
PRRT1B	A0A1B0GWB2	0	0.0000	0	0.0000	0	0.0000	0	0.0000	0	0.0000	3	0.2143	3	0.0140
SPATC1L	Q9H0A9	0	0.0000	0	0.0000	0	0.0000	0	0.0000	0	0.0000	3	0.2143	3	0.0140
DACH1	Q9UI36	0	0.0000	0	0.0000	0	0.0000	1	0.2500	0	0.0000	2	0.1429	3	0.0140
SLC7A14	Q8TBB6	0	0.0000	0	0.0000	0	0.0000	0	0.0000	0	0.0000	3	0.2143	3	0.0140
SEMA3D	O95025	0	0.0000	0	0.0000	0	0.0000	0	0.0000	0	0.0000	3	0.2143	3	0.0140
HK2	P52789	1	0.0049	0	0.0000	0	0.0000	0	0.0000	0	0.0000	2	0.1429	3	0.0140
FSTL5	Q8N475	0	0.0000	0	0.0000	0	0.0000	0	0.0000	0	0.0000	3	0.2143	3	0.0140
TCF4	P15884	1	0.0049	0	0.0000	0	0.0000	0	0.0000	0	0.0000	2	0.1429	3	0.0140
GPRASP1	Q5JY77	0	0.0000	0	0.0000	0	0.0000	0	0.0000	0	0.0000	3	0.2143	3	0.0140
DRAXIN	Q8NBI3	0	0.0000	0	0.0000	0	0.0000	0	0.0000	0	0.0000	3	0.2143	3	0.0140
EYS	Q5T1H1	0	0.0000	0	0.0000	0	0.0000	0	0.0000	0	0.0000	3	0.2143	3	0.0140
ZNF619	Q8N2I2	1	0.0049	0	0.0000	0	0.0000	0	0.0000	0	0.0000	2	0.1429	3	0.0140
CRYZ	Q08257	0	0.0000	0	0.0000	0	0.0000	0	0.0000	0	0.0000	3	0.2143	3	0.0140
GPR149	Q86SP6	0	0.0000	0	0.0000	0	0.0000	0	0.0000	0	0.0000	3	0.2143	3	0.0140
IGFBPL1	Q8WX77	0	0.0000	0	0.0000	0	0.0000	0	0.0000	0	0.0000	3	0.2143	3	0.0140

GRID1	Q9ULK0	0	0.0000	0	0.0000	0	0.0000	0	0.0000	0	0.0000	3	0.2143	3	0.0140
RTL9	Q8NET4	0	0.0000	0	0.0000	0	0.0000	0	0.0000	0	0.0000	3	0.2143	3	0.0140
SMOC1	Q9H4F8	0	0.0000	0	0.0000	0	0.0000	0	0.0000	0	0.0000	3	0.2143	3	0.0140
PHOX2A	O14813	0	0.0000	0	0.0000	0	0.0000	0	0.0000	0	0.0000	3	0.2143	3	0.0140
EVA1C	P58658	0	0.0000	0	0.0000	0	0.0000	0	0.0000	0	0.0000	3	0.2143	3	0.0140
CXXC5	Q7LFL8	1	0.0049	0	0.0000	0	0.0000	0	0.0000	0	0.0000	2	0.1429	3	0.0140
RGPD1	P0DJD0	0	0.0000	0	0.0000	0	0.0000	0	0.0000	0	0.0000	3	0.2143	3	0.0140
SYT3	Q9BQG1	0	0.0000	0	0.0000	0	0.0000	0	0.0000	0	0.0000	3	0.2143	3	0.0140
NRN1	Q9NPD7	1	0.0049	0	0.0000	0	0.0000	0	0.0000	0	0.0000	2	0.1429	3	0.0140
GNAO1	P09471	1	0.0049	0	0.0000	0	0.0000	0	0.0000	0	0.0000	2	0.1429	3	0.0140
CNTNAP 4	Q9C0A0	0	0.0000	0	0.0000	0	0.0000	0	0.0000	0	0.0000	3	0.2143	3	0.0140
ANKRD1	Q15327	1	0.0049	0	0.0000	0	0.0000	0	0.0000	0	0.0000	2	0.1429	3	0.0140
NWD2	Q9ULI1	0	0.0000	0	0.0000	0	0.0000	0	0.0000	0	0.0000	3	0.2143	3	0.0140
ZNF506	Q5JVG8	1	0.0049	0	0.0000	0	0.0000	0	0.0000	0	0.0000	2	0.1429	3	0.0140
FUT11	Q495W5	1	0.0049	0	0.0000	0	0.0000	0	0.0000	0	0.0000	2	0.1429	3	0.0140
PCDH10	Q9P2E7	0	0.0000	0	0.0000	0	0.0000	0	0.0000	0	0.0000	3	0.2143	3	0.0140
LYPD1	Q8N2G4	1	0.0049	0	0.0000	0	0.0000	0	0.0000	0	0.0000	2	0.1429	3	0.0140
TLN2	Q9Y4G6	0	0.0000	0	0.0000	0	0.0000	0	0.0000	0	0.0000	3	0.2143	3	0.0140
MKX	Q8IYA7	0	0.0000	0	0.0000	0	0.0000	0	0.0000	0	0.0000	3	0.2143	3	0.0140
NBPF26	B4DH59	0	0.0000	0	0.0000	0	0.0000	0	0.0000	0	0.0000	3	0.2143	3	0.0140
MAF	O75444	1	0.0049	0	0.0000	0	0.0000	0	0.0000	0	0.0000	2	0.1429	3	0.0140
STMN2	Q93045	1	0.0049	0	0.0000	0	0.0000	0	0.0000	0	0.0000	2	0.1429	3	0.0140
ZNF736	B4DX44	1	0.0049	0	0.0000	0	0.0000	0	0.0000	0	0.0000	2	0.1429	3	0.0140
TBX18	O95935	0	0.0000	0	0.0000	0	0.0000	0	0.0000	0	0.0000	3	0.2143	3	0.0140
GALNTL 6	Q49A17	1	0.0049	0	0.0000	0	0.0000	0	0.0000	0	0.0000	2	0.1429	3	0.0140
GPR137 C	Q8N3F9	0	0.0000	0	0.0000	0	0.0000	0	0.0000	0	0.0000	3	0.2143	3	0.0140

MYT1	Q01538	0	0.0000	0	0.0000	0	0.0000	0	0.0000	0	0.0000	3	0.2143	3	0.0140
TMOD1	P28289	1	0.0049	0	0.0000	0	0.0000	0	0.0000	0	0.0000	2	0.1429	3	0.0140
TMEM196	Q5HYL7	0	0.0000	0	0.0000	0	0.0000	0	0.0000	0	0.0000	3	0.2143	3	0.0140
KLF9	Q13886	0	0.0000	0	0.0000	0	0.0000	0	0.0000	0	0.0000	3	0.2143	3	0.0140
ANTXR1	Q9H6X2	0	0.0000	0	0.0000	0	0.0000	0	0.0000	0	0.0000	3	0.2143	3	0.0140
GCNT2	Q8N0V5	0	0.0000	0	0.0000	0	0.0000	0	0.0000	0	0.0000	3	0.2143	3	0.0140
RIMBP2	O15034	0	0.0000	0	0.0000	0	0.0000	0	0.0000	0	0.0000	3	0.2143	3	0.0140
NRSN1	Q8IZ57	0	0.0000	0	0.0000	0	0.0000	0	0.0000	0	0.0000	3	0.2143	3	0.0140
ZP3	P21754	1	0.0049	0	0.0000	0	0.0000	0	0.0000	0	0.0000	2	0.1429	3	0.0140
NSDHL	Q15738	1	0.0049	0	0.0000	0	0.0000	0	0.0000	0	0.0000	2	0.1429	3	0.0140
CPLX2	Q6PUV4	0	0.0000	0	0.0000	0	0.0000	0	0.0000	0	0.0000	3	0.2143	3	0.0140
PRSS12	P56730	0	0.0000	0	0.0000	0	0.0000	0	0.0000	0	0.0000	3	0.2143	3	0.0140
BNIP3L	O60238	1	0.0049	0	0.0000	0	0.0000	0	0.0000	0	0.0000	2	0.1429	3	0.0140
KLHL1	Q9NR64	0	0.0000	0	0.0000	0	0.0000	0	0.0000	0	0.0000	2	0.1429	2	0.0093
TMEM132C	Q8N3T6	0	0.0000	0	0.0000	0	0.0000	0	0.0000	0	0.0000	2	0.1429	2	0.0093
NAP1L5	Q96NT1	0	0.0000	0	0.0000	0	0.0000	0	0.0000	0	0.0000	2	0.1429	2	0.0093
FRMPD1	Q5SYB0	0	0.0000	0	0.0000	0	0.0000	0	0.0000	0	0.0000	2	0.1429	2	0.0093
LMX1B	O60663	0	0.0000	0	0.0000	0	0.0000	0	0.0000	0	0.0000	2	0.1429	2	0.0093
CTNND2	Q9UQB3	0	0.0000	0	0.0000	0	0.0000	0	0.0000	0	0.0000	2	0.1429	2	0.0093
CPXM2	Q8N436	0	0.0000	0	0.0000	0	0.0000	0	0.0000	0	0.0000	2	0.1429	2	0.0093
FEV	Q99581	0	0.0000	0	0.0000	0	0.0000	0	0.0000	0	0.0000	2	0.1429	2	0.0093
TP53TG3F	Q9ULZ0	0	0.0000	0	0.0000	0	0.0000	0	0.0000	0	0.0000	2	0.1429	2	0.0093
PHOX2B	Q99453	0	0.0000	0	0.0000	0	0.0000	0	0.0000	0	0.0000	2	0.1429	2	0.0093
BNIP3	Q12983	0	0.0000	0	0.0000	0	0.0000	0	0.0000	0	0.0000	2	0.1429	2	0.0093
CHD5	Q8TDI0	0	0.0000	0	0.0000	0	0.0000	0	0.0000	0	0.0000	2	0.1429	2	0.0093

HEPACAM2	A8MVW5	0	0.0000	0	0.0000	0	0.0000	0	0.0000	0	0.0000	2	0.1429	2	0.0093
SYT13	Q7L8C5	0	0.0000	0	0.0000	0	0.0000	0	0.0000	0	0.0000	2	0.1429	2	0.0093
TMEM255A	Q5JRV8	0	0.0000	0	0.0000	0	0.0000	0	0.0000	0	0.0000	2	0.1429	2	0.0093
MMP24OS	A0A0U1RRL7	0	0.0000	0	0.0000	0	0.0000	0	0.0000	0	0.0000	2	0.1429	2	0.0093
PCDHGB4	Q9UN71	0	0.0000	0	0.0000	0	0.0000	0	0.0000	0	0.0000	2	0.1429	2	0.0093
STUM	Q69YW2	0	0.0000	0	0.0000	0	0.0000	0	0.0000	0	0.0000	2	0.1429	2	0.0093
PTPRT	O14522	0	0.0000	0	0.0000	0	0.0000	0	0.0000	0	0.0000	2	0.1429	2	0.0093
LAMP5	Q9UJQ1	0	0.0000	0	0.0000	0	0.0000	0	0.0000	0	0.0000	2	0.1429	2	0.0093
ZNF428	Q96B54	0	0.0000	0	0.0000	0	0.0000	0	0.0000	0	0.0000	2	0.1429	2	0.0093
PLXDC2	Q6UX71	0	0.0000	0	0.0000	0	0.0000	0	0.0000	0	0.0000	2	0.1429	2	0.0093
FAM162A	Q96A26	0	0.0000	0	0.0000	0	0.0000	0	0.0000	0	0.0000	2	0.1429	2	0.0093
SERP2	Q8N6R1	0	0.0000	0	0.0000	0	0.0000	0	0.0000	0	0.0000	2	0.1429	2	0.0093
DIRAS1	O95057	0	0.0000	0	0.0000	0	0.0000	0	0.0000	0	0.0000	2	0.1429	2	0.0093
CBLN4	Q9NTU7	0	0.0000	0	0.0000	0	0.0000	0	0.0000	0	0.0000	2	0.1429	2	0.0093
ADGRG5	Q8IZF4	0	0.0000	0	0.0000	0	0.0000	0	0.0000	0	0.0000	2	0.1429	2	0.0093
NEUROD6	Q96NK8	0	0.0000	0	0.0000	0	0.0000	0	0.0000	0	0.0000	2	0.1429	2	0.0093
SMIM31	A0A1B0GVY4	0	0.0000	0	0.0000	0	0.0000	0	0.0000	0	0.0000	2	0.1429	2	0.0093
TRIM58	Q8NG06	0	0.0000	0	0.0000	0	0.0000	0	0.0000	0	0.0000	2	0.1429	2	0.0093
CAMK2N1	Q7Z7J9	0	0.0000	0	0.0000	0	0.0000	0	0.0000	0	0.0000	2	0.1429	2	0.0093
TMC3	Q7Z5M5	0	0.0000	0	0.0000	0	0.0000	0	0.0000	0	0.0000	2	0.1429	2	0.0093
ZNF572	Q7Z3I7	0	0.0000	0	0.0000	0	0.0000	0	0.0000	0	0.0000	2	0.1429	2	0.0093
POU3F4	P49335	0	0.0000	0	0.0000	0	0.0000	0	0.0000	0	0.0000	2	0.1429	2	0.0093
MAP10	Q9P2G4	0	0.0000	0	0.0000	0	0.0000	0	0.0000	0	0.0000	2	0.1429	2	0.0093
STC1	P52823	0	0.0000	0	0.0000	0	0.0000	0	0.0000	0	0.0000	2	0.1429	2	0.0093

FSTL4	Q6MZW2	0	0.0000	0	0.0000	0	0.0000	0	0.0000	0	0.0000	2	0.1429	2	0.0093
MYOM2	P54296	0	0.0000	0	0.0000	0	0.0000	0	0.0000	0	0.0000	2	0.1429	2	0.0093
TMEM163	Q8TC26	0	0.0000	0	0.0000	0	0.0000	0	0.0000	0	0.0000	2	0.1429	2	0.0093
LMO4	P61968	0	0.0000	0	0.0000	0	0.0000	0	0.0000	0	0.0000	2	0.1429	2	0.0093
R3HDML	Q9H3Y0	0	0.0000	0	0.0000	0	0.0000	0	0.0000	0	0.0000	2	0.1429	2	0.0093
AFF2	P51816	0	0.0000	0	0.0000	0	0.0000	0	0.0000	0	0.0000	2	0.1429	2	0.0093
RTN1	Q16799	0	0.0000	0	0.0000	0	0.0000	0	0.0000	0	0.0000	2	0.1429	2	0.0093
TMEM42	Q69YG0	0	0.0000	0	0.0000	0	0.0000	0	0.0000	0	0.0000	2	0.1429	2	0.0093
TCERG1L	Q5VWI1	0	0.0000	0	0.0000	0	0.0000	0	0.0000	0	0.0000	2	0.1429	2	0.0093
AJAP1	Q9UKB5	0	0.0000	0	0.0000	0	0.0000	0	0.0000	0	0.0000	2	0.1429	2	0.0093
POMZP3	Q6PJE2	0	0.0000	0	0.0000	0	0.0000	0	0.0000	0	0.0000	2	0.1429	2	0.0093
LYPD6	Q86Y78	0	0.0000	0	0.0000	0	0.0000	0	0.0000	0	0.0000	2	0.1429	2	0.0093
POU6F2	P78424	0	0.0000	0	0.0000	0	0.0000	0	0.0000	0	0.0000	2	0.1429	2	0.0093
TUSC1	Q2TAM9	0	0.0000	0	0.0000	0	0.0000	0	0.0000	0	0.0000	2	0.1429	2	0.0093
ZXDA	P98168	0	0.0000	0	0.0000	0	0.0000	0	0.0000	0	0.0000	2	0.1429	2	0.0093
PCDHGA7	Q9Y5G6	0	0.0000	0	0.0000	0	0.0000	0	0.0000	0	0.0000	2	0.1429	2	0.0093
TCEAL5	Q5H9L2	0	0.0000	0	0.0000	0	0.0000	0	0.0000	0	0.0000	2	0.1429	2	0.0093
PCDHGA3	Q9Y5H0	0	0.0000	0	0.0000	0	0.0000	0	0.0000	0	0.0000	2	0.1429	2	0.0093
PCDHGA10	Q9Y5H3	0	0.0000	0	0.0000	0	0.0000	0	0.0000	0	0.0000	2	0.1429	2	0.0093
PCDHGA11	Q9Y5H2	0	0.0000	0	0.0000	0	0.0000	0	0.0000	0	0.0000	2	0.1429	2	0.0093
ILDR2	Q71H61	0	0.0000	0	0.0000	0	0.0000	0	0.0000	0	0.0000	2	0.1429	2	0.0093
CBLN2	Q8IUK8	0	0.0000	0	0.0000	0	0.0000	0	0.0000	0	0.0000	2	0.1429	2	0.0093
PCDHA5	Q9Y5H7	0	0.0000	0	0.0000	0	0.0000	0	0.0000	0	0.0000	2	0.1429	2	0.0093
TOGARAM2	Q6ZUX3	0	0.0000	0	0.0000	0	0.0000	0	0.0000	0	0.0000	2	0.1429	2	0.0093

LDOC1	O95751	0	0.0000	0	0.0000	0	0.0000	0	0.0000	0	0.0000	2	0.1429	2	0.0093
ZNF229	Q9UJW7	0	0.0000	0	0.0000	0	0.0000	0	0.0000	0	0.0000	2	0.1429	2	0.0093
TENM1	Q9UKZ4	0	0.0000	0	0.0000	0	0.0000	0	0.0000	0	0.0000	2	0.1429	2	0.0093
EBF3	Q9H4W6	0	0.0000	0	0.0000	0	0.0000	0	0.0000	0	0.0000	2	0.1429	2	0.0093
RALYL	Q86SE5	0	0.0000	0	0.0000	0	0.0000	0	0.0000	0	0.0000	2	0.1429	2	0.0093
MAGEH1	Q9H213	0	0.0000	0	0.0000	0	0.0000	0	0.0000	0	0.0000	2	0.1429	2	0.0093

Supplementary Table 3. Previously published studies using midbrain organoids integrated to create the PD-KG graph database.

Mutation	Study	Main findings	Cell lines	No. of organoid differentiation
**LRRK2-G2019S**	Smits et al., 2019 (imaging)<br><br>Zagare & Barmpa et al., 2022 (scRNAseq)	- Decreased number and complexity of midbrain dopaminergic neurons<br><br>- Neurodevelopmental defects of midbrain dopaminergic neurons	2WT, 2WT-GC, 2PD, 2PD-GC (imaging)<br><br>1WT, 1WT-GC (scRNAseq)	Data from 5 organoid differentiation (imaging)<br><br>Data from 30 organoids (1 differentiation) (scRNAseq)
**3xSNCA**	Modamio et al., 2021	- Increased levels of α-synuclein and, phosphorylated (pSer129) α-synuclein<br>- Impaired synaptic function<br>- Loss of dopaminergic neurons over time	3WT, 2PD (imaging)<br><br>1WT, 1PD (bulkRNAseq)	N/A (imaging)<br><br>Organoids pooled from 3 differentiations (bulkRNAseq)
**GBA-N370S**	Rosety et al., 2023	- Neurogenesis defects<br>- Altered lipid metabolism,<br>- Reduced GCase activity<br>- Reduced levels of dopaminergic neurons	3WT, 3PD (imaging)<br><br>2WT,2PD (bulkRNAseq)	>=3 differentiations (imaging)<br><br>8 Organoids pooled from 3 differentiations (bulkRNAseq)
**MIRO1-R272Q**	Chemla et al., 2023	- Mitochondrial impairments<br>- Increased levels of α-synuclein<br>- Dopaminergic neuron death	3WT,1PD,1PD-GC (imaging)<br><br>1WT, 1PD, 1PD-GC (scRNAseq)	>=3 differentiations (imaging)<br><br>Data from 30 organoids (1 differentiation) (scRNAseq)

WT: Wild-type/healthy control

GC: gene-corrected line/isogenic control

scRNAseq: single-cell RNA sequencing

bulkRNAseq: bulk RNA sequencing

Supplementary Table 4. Comparison of the PD-KG with other knowledge graphs for neurodegenerative diseases.

	**PD-KG**	**NeuroMMSig (10.1093/bioinformatics/btx399)**	**AlzKB (10.2196/46777)**
**Neurodegenerative disease**	Parkinson's	Parkinson's; Alzheimer's	Alzheimer's
**Focus**	Experimental data-centric	Knowledge-centric	Knowledge-centric
**Building process**	Experimental data contextualized with data from public resources	Literature review of causal mechanisms enriched with data from public resources	Ontology built from expert knowledge and populated with data from public resources
**Concepts**	CoreProtein (13); Gene (440); CellLine (30) CellLineTimePoint (127); Feature (1); Normalised Feature (13); Disease (575); Pathway (670); Drug (445); NeighbourgProtein (1296)	NA	Gene (62,407); Drug (35,063); BiologicalProcess (11,381); Pathway (4570); MolecularFunction (2884); CellularComponent (1391); Symptom (438); BodyPart (402); DrugClass (345); Disease (20)
**Size**	3,612 entities; 8,512 relationships	NA	118,902 entities; 1,309,527 relationships
**Contained experimental data types**	Protein imaging (existing and new datasets); RNAseq (existing and new datasets)	GWAS variants (GWAS Catalog and GWASdb); imaging features; co-expression network analysis (NeuroTransDB)	Tissue specific gene expression data (Bgee and TISSUES); Chemical toxicity data (EPA DSSTox and EPA ACToR); Human differential gene expression data (LINCS L1000)
**Technology**	Neo4j	Neo4j	Neo4j

Supplementary Table 5. List of 25 genes shared between at least two RNA sequencing experiments of monogenic PD patient specific midbrain organoid studies.

Gene symbol	No. of shared experiments	Shared experiments
FTL	2	LRRK2, MIRO1
PTPRD	2	LRRK2, MIRO1
MAB21L2	2	LRRK2, GBA
GAD1	2	LRRK2, MIRO1
GRIA2	2	LRRK2, GBA
CHL1	2	LRRK2, MIRO1
TUBA1A	2	LRRK2, MIRO1
ACTB	2	LRRK2, MIRO1
ACTG1	2	LRRK2, MIRO1
ENO1	2	LRRK2, MIRO1
PAX5	2	LRRK2, SNCA
MORF4L2	2	LRRK2, MIRO1
MAB21L1	2	LRRK2, GBA
IGFBP2	2	LRRK2, MIRO1
EN1	2	LRRK2, MIRO1
SLC17A6	2	LRRK2, MIRO1
TUBB2B	2	LRRK2, MIRO1
OTP	2	LRRK2, SNCA
ROBO2	2	LRRK2, MIRO1
PEG10	2	LRRK2, MIRO1
EPHA5	2	GBA, MIRO1
TCEAL7	2	GBA, MIRO1
TMEM132B	2	GBA, SNCA
CBLN1	2	SNCA, MIRO1
DPP6	2	SNCA, MIRO1

Supplementary Table 6. Expression pattern of the 25 shared genes between at least two experiments in PD samples vs healthy controls (CTRL).

Gene symbol	Expression in PD vs CTRL	Shared experiments
MAB21L2	DOWN	LRRK2, GBA
GAD1	DOWN	LRRK2, MIRO1
GRIA2	DOWN	LRRK2, GBA
TUBA1A	UP	LRRK2, MIRO1
ACTB	UP	LRRK2, MIRO1
ACTG1	UP	LRRK2, MIRO1
MAB21L1	DOWN	LRRK2, GBA
TUBB2B	UP	LRRK2, MIRO1
PEG10	UP	LRRK2, MIRO1
TCEAL7	DOWN	GBA, MIRO1
CBLN1	UP	SNCA, MIRO1
DPP6	UP	SNCA, MIRO1
FTL	UP, DOWN	LRRK2, MIRO1
PTPRD	DOWN, UP	LRRK2, MIRO1
CHL1	DOWN, UP	LRRK2, MIRO1
ENO1	UP, DOWN	LRRK2, MIRO1
PAX5	DOWN, UP	LRRK2, SNCA
MORF4L2	UP, DOWN	LRRK2, MIRO1
IGFBP2	UP, DOWN	LRRK2, MIRO1
EN1	UP, DOWN	LRRK2, MIRO1
SLC17A6	DOWN, UP	LRRK2, MIRO1
OTP	DOWN, UP	LRRK2, SNCA
ROBO2	DOWN, UP	LRRK2, MIRO1
EPHA5	DOWN, UP	GBA, MIRO1
TMEM132B	DOWN, UP	GBA, SNCA

Supplementary Table 7. Reactome pathways associated with the most connected six out of 25 genes shared between genetic PD datasets.

Pathway	Gene symbol	No. of genes
"RECYCLING PATHWAY OF L1"	["ACTB", "TUBA1A", "ACTG1", "TUBB2B"]	4
"TRANSLOCATION OF SLC2A4 (GLUT4) TO THE PLASMA MEMBRANE"	["ACTG1", "TUBB2B", "ACTB", "TUBA1A"]	4
"RHO GTPASES ACTIVATE IQGAPS"	["ACTG1", "TUBB2B", "TUBA1A", "ACTB"]	4
"RHO GTPASES ACTIVATE FORMINS"	["ACTB", "TUBB2B", "TUBA1A", "ACTG1"]	4
"EPH-EPHRIN MEDIATED REPULSION OF CELLS"	["EPHA5", "ACTB", "ACTG1"]	3
"PREFOLDIN MEDIATED TRANSFER OF SUBSTRATE TO CCT/TRIC"	["TUBB2B", "TUBA1A", "ACTB"]	3
"RECRUITMENT OF NUMA TO MITOTIC CENTROSOMES"	["TUBB2B", "TUBA1A"]	2
"VEGFA-VEGFR2 PATHWAY"	["ACTG1", "ACTB"]	2
"CELL-EXTRACELLULAR MATRIX INTERACTIONS"	["ACTB", "ACTG1"]	2
"ASSEMBLY AND CELL SURFACE PRESENTATION OF NMDA RECEPTORS"	["TUBA1A", "TUBB2B"]	2
"HCMV EARLY EVENTS"	["TUBA1A", "TUBB2B"]	2
"INTERACTION BETWEEN L1 AND ANKYRINS"	["ACTG1", "ACTB"]	2
"ACTIVATION OF AMPK DOWNSTREAM OF NMDARS"	["TUBB2B", "TUBA1A"]	2
"SEPARATION OF SISTER CHROMATIDS"	["TUBA1A", "TUBB2B"]	2
"SIGNALING DOWNSTREAM OF RAS MUTANTS"	["ACTB", "ACTG1"]	2
"CLATHRIN-MEDIATED ENDOCYTOSIS"	["ACTG1", "ACTB"]	2
"SIGNALING BY RAF1 MUTANTS"	["ACTG1", "ACTB"]	2
"SEALING OF THE NUCLEAR ENVELOPE (NE) BY ESCRT-III"	["TUBB2B", "TUBA1A"]	2
"ADHERENS JUNCTIONS INTERACTIONS"	["ACTB", "ACTG1"]	2
"SIGNALING BY BRAF AND RAF1 FUSIONS"	["ACTG1", "ACTB"]	2
"PARADOXICAL ACTIVATION OF RAF SIGNALING BY KINASE INACTIVE BRAF"	["ACTG1", "ACTB"]	2
"THE ROLE OF GTSE1 IN G2/M PROGRESSION AFTER G2 CHECKPOINT"	["TUBA1A", "TUBB2B"]	2
"CILIUM ASSEMBLY"	["TUBA1A", "TUBB2B"]	2
"SIGNALING BY MODERATE KINASE ACTIVITY BRAF MUTANTS"	["ACTB", "ACTG1"]	2

**"SIGNALING BY HIGH-KINASE ACTIVITY BRAF MUTANTS"**	["ACTG1", "ACTB"]	2
**"EPHB-MEDIATED FORWARD SIGNALING"**	["ACTB", "ACTG1"]	2
**"REGULATION OF ACTIN DYNAMICS FOR PHAGOCYTIC CUP FORMATION"**	["ACTG1", "ACTB"]	2
**"COPI-INDEPENDENT GOLGI-TO-ER RETROGRADE TRAFFIC"**	["TUBA1A", "TUBB2B"]	2
**"COPI-DEPENDENT GOLGI-TO-ER RETROGRADE TRAFFIC"**	["TUBA1A", "TUBB2B"]	2
**"KINESINS"**	["TUBB2B", "TUBA1A"]	2
**"AGGREPHAGY"**	["TUBB2B", "TUBA1A"]	2
**"MAP2K AND MAPK ACTIVATION"**	["ACTG1", "ACTB"]	2
**"MHC CLASS II ANTIGEN PRESENTATION"**	["TUBA1A", "TUBB2B"]	2
**"RESOLUTION OF SISTER CHROMATID COHESION"**	["TUBB2B", "TUBA1A"]	2
**"MITOTIC PROMETAPHASE"**	["TUBB2B", "TUBA1A"]	2
**"PKR-MEDIATED SIGNALING"**	["TUBA1A", "TUBB2B"]	2
**"INTRAFLAGELLAR TRANSPORT"**	["TUBA1A", "TUBB2B"]	2
**"SENSORY PROCESSING OF SOUND BY INNER HAIR CELLS OF THE COCHLEA"**	["ACTB", "ACTG1"]	2
**"SENSORY PROCESSING OF SOUND BY OUTER HAIR CELLS OF THE COCHLEA"**	["ACTG1", "ACTB"]	2
**"FCGR3A-MEDIATED PHAGOCYTOSIS"**	["ACTG1", "ACTB"]	2
**"GAP JUNCTION DEGRADATION"**	["ACTG1", "ACTB"]	2
**"HEDGEHOG 'OFF' STATE"**	["TUBA1A", "TUBB2B"]	2
**"GAP JUNCTION ASSEMBLY"**	["TUBB2B", "TUBA1A"]	2
**"FORMATION OF ANNULAR GAP JUNCTIONS"**	["ACTB", "ACTG1"]	2
**"MICROTUBULE-DEPENDENT TRAFFICKING OF CONNEXONS FROM GOLGI TO THE PLASMA MEMBRANE"**	["TUBB2B", "TUBA1A"]	2
**"CARBOXYTERMINAL POST-TRANSLATIONAL MODIFICATIONS OF TUBULIN"**	["TUBA1A", "TUBB2B"]	2
**"POST-CHAPERONIN TUBULIN FOLDING PATHWAY"**	["TUBB2B", "TUBA1A"]	2
**"HATS ACETYLATE HISTONES"**	["ACTB", "MORF4L2"]	2
**"COPI-MEDIATED ANTEROGRADE TRANSPORT"**	["TUBB2B", "TUBA1A"]	2
**"EML4 AND NUDC IN MITOTIC SPINDLE FORMATION"**	["TUBA1A", "TUBB2B"]	2

**"HSP90 CHAPERONE CYCLE FOR STEROID HORMONE RECEPTORS (SHR) IN THE PRESENCE OF LIGAND"**	["TUBB2B", "TUBA1A"]	2
**"RHO GTPASES ACTIVATE WASPS AND WAVES"**	["ACTB", "ACTG1"]	2
**"FORMATION OF TUBULIN FOLDING INTERMEDIATES BY CCT/TRIC"**	["TUBA1A", "TUBB2B"]	2
**"RHOBTB2 GTPASE CYCLE"**	["ACTG1"]	1
**"B-WICH COMPLEX POSITIVELY REGULATES RRNA EXPRESSION"**	["ACTB"]	1
**"REGULATION OF MITF-M-DEPENDENT GENES INVOLVED IN PIGMENTATION"**	["ACTB"]	1
**"FOLDING OF ACTIN BY CCT/TRIC"**	["ACTB"]	1
**"FACTORS INVOLVED IN MEGAKARYOCYTE DEVELOPMENT AND PLATELET PRODUCTION"**	["ACTB"]	1
**"ANCHORING OF THE BASAL BODY TO THE PLASMA MEMBRANE"**	["TUBA1A"]	1
**"LOSS OF PROTEINS REQUIRED FOR INTERPHASE MICROTUBULE ORGANIZATION FROM THE CENTROSOME"**	["TUBA1A"]	1
**"EPH-EPHRIN SIGNALING"**	["EPHA5"]	1
**"RHOF GTPASE CYCLE"**	["ACTB"]	1
**"RECRUITMENT OF MITOTIC CENTROSOME PROTEINS AND COMPLEXES"**	["TUBA1A"]	1
**"LOSS OF NLP FROM MITOTIC CENTROSOMES"**	["TUBA1A"]	1
**"EPHA-MEDIATED GROWTH CONE COLLAPSE"**	["EPHA5"]	1
**"DNA DAMAGE RECOGNITION IN GG-NER"**	["ACTB"]	1
**"AURKA ACTIVATION BY TPX2"**	["TUBA1A"]	1
**"REGULATION OF PLK1 ACTIVITY AT G2/M TRANSITION"**	["TUBA1A"]	1
**"UCH PROTEINASES"**	["ACTB"]	1

Supplementary Table 8. List of Core Proteins. Proteins that were immunostained in different experiments for high-content imaging analysis.

No.	Gene symbol	Uniprot Id	Protein name	Experiments
**1**	CNP	P09543	2',3'-cyclic-nucleotide 3'-phosphodiesterase	GBA
**2**	DCX	O43602	Neuronal migration protein doublecortin	GBA
**3**	FOXA2	Q9Y261	Hepatocyte nuclear factor 3-beta	GBA, LRRK2
**4**	GFAP	P14136	Glial fibrillary acidic protein	SNCA, MIRO1, IPD, GBA
**5**	MAP2	P11137	Microtubule-associated protein 2	SNCA, MIRO1, IPD, GBA
**6**	MKI67	P46013	Proliferation marker protein Ki-67	IPD, GBA
**7**	NES	P48681	Nestin	GBA
**8**	OLIG2	Q13516	Oligodendrocyte transcription factor 2	GBA
**9**	PAX6	P26367	Paired box protein Pax-6	IPD
**10**	S100B	P04271	Protein S100-B	SNCA, MIRO1, IPD, GBA
**11**	SOX2	P48431	Transcription factor SOX-2	GBA, IPD
**12**	TH	P07101	Tyrosine hydroxylase	SNCA, MIRO1, IPD, GBA, LRRK2
**13**	TUBB3/TUJ1	Q13509	Tubulin beta-3 chain	[SNCA, MIRO1, IPD, GBA]

Supplementary Table 9. Shared pathways between core proteins and significant differential expressed genes in individual datasets.

Reactome Pathway name	Core proteins	Genes	Experiment
CARBOXYTERMINAL POST-TRANSLATIONAL MODIFICATIONS OF TUBULIN	TUBB3	VASH2	GBA
CARBOXYTERMINAL POST-TRANSLATIONAL MODIFICATIONS OF TUBULIN	TUBB3	TUBA1A, TUBB2B	MIRO1
HEDGEHOG 'OFF' STATE	TUBB3	ADCY1	GBA
HEDGEHOG 'OFF' STATE	TUBB3	TUBA1A, TUBB2B	MIRO1
RECYCLING PATHWAY OF L1	TUBB3	L1CAM	GBA
RECYCLING PATHWAY OF L1	TUBB3	ACTB, TUBA1A, ACTG1, TUBB2B	MIRO1
SEPARATION OF SISTER CHROMATIDS	TUBB3	PSMD5	SNCA
SEPARATION OF SISTER CHROMATIDS	TUBB3	TUBA1A, TUBB2B	MIRO1
THE ROLE OF GTSE1 IN G2/M PROGRESSION AFTER G2 CHECKPOINT	TUBB3	PSMD5	SNCA
THE ROLE OF GTSE1 IN G2/M PROGRESSION AFTER G2 CHECKPOINT	TUBB3	TUBA1A, CDKN1A, TUBB2B	MIRO1
MHC CLASS II ANTIGEN PRESENTATION	TUBB3	CTSF	SNCA
MHC CLASS II ANTIGEN PRESENTATION	TUBB3	TUBA1A, TUBB2B	MIRO1
NUCLEAR SIGNALING BY ERBB4	GFAP, S100B	GFAP	SNCA
NUCLEAR SIGNALING BY ERBB4	GFAP, S100B	STMN1	MIRO1
CATECHOLAMINE BIOSYNTHESIS	TH	TH	GBA
CHAPERONE MEDIATED AUTOPHAGY	GFAP	GFAP	SNCA
FORMATION OF THE ANTERIOR NEURAL PLATE	SOX2	POU3F1	GBA
FORMATION OF THE POSTERIOR NEURAL PLATE	SOX2	POU3F1	GBA
INTERLEUKIN-4 AND INTERLEUKIN-13 SIGNALING	SOX2	HGF	GBA
COPI-DEPENDENT GOLGI-TO-ER RETROGRADE TRAFFIC	TUBB3	TUBA1A, TUBB2B	MIRO1
RECRUITMENT OF NUMA TO MITOTIC CENTROSOMES	TUBB3	TUBB2B, TUBA1A, TUBB	MIRO1
CILIUM ASSEMBLY	TUBB3	TUBA1A, TUBB2B	MIRO1
INTRAFLAGELLAR TRANSPORT	TUBB3	TUBA1A, TUBB2B	MIRO1
TRANSLOCATION OF SLC2A4 (GLUT4) TO THE PLASMA MEMBRANE	TUBB3	ACTG1, TUBB2B, ACTB, TUBA1A	MIRO1
HSP90 CHAPERONE CYCLE FOR STEROID HORMONE RECEPTORS (SHR) IN THE PRESENCE OF LIGAND	TUBB3	TUBB2B, TUBA1A	MIRO1
RHO GTPASES ACTIVATE FORMINS	TUBB3	ACTB, TUBB2B, TUBA1A, ACTG1	MIRO1
ACTIVATION OF AMPK DOWNSTREAM OF NMDARS	TUBB3	TUBB2B, TUBA1A	MIRO1
SEALING OF THE NUCLEAR ENVELOPE (NE) BY ESCRT-III	TUBB3	TUBB2B, TUBA1A	MIRO1
RHO GTPASES ACTIVATE IQGAPS	TUBB3	ACTG1, TUBB2B, TUBA1A, ACTB	MIRO1
FORMATION OF TUBULIN FOLDING INTERMEDIATES BY CCT/TRIC	TUBB3	TUBA1A, TUBB2B	MIRO1
MICROTUBULE-DEPENDENT TRAFFICKING OF CONNEXONS FROM GOLGI TO THE PLASMA MEMBRANE	TUBB3	TUBB2B, TUBA1A	MIRO1
COPI-INDEPENDENT GOLGI-TO-ER RETROGRADE TRAFFIC	TUBB3	TUBA1A, TUBB2B	MIRO1
AGGREPHAGY	TUBB3	TUBB2B, TUBA1A	MIRO1
HCMV EARLY EVENTS	TUBB3	TUBA1A, TUBB2B	MIRO1
EML4 AND NUDC IN MITOTIC SPINDLE FORMATION	TUBB3	TUBA1A, TUBB2B	MIRO1
RESOLUTION OF SISTER CHROMATID COHESION	TUBB3	TUBB2B, TUBA1A	MIRO1
PKR-MEDIATED SIGNALING	TUBB3	TUBA1A, TUBB2B	MIRO1
MITOTIC PROMETAPHASE	TUBB3	TUBB2B, TUBA1A	MIRO1
COPI-MEDIATED ANTEROGRADE TRANSPORT	TUBB3	TUBB2B, TUBA1A	MIRO1

PREFOLDIN MEDIATED TRANSFER OF SUBSTRATE TO CCT/TRIC	TUBB3	TUBB2B, TUBA1A, ACTB	MIRO1
ASSEMBLY AND CELL SURFACE PRESENTATION OF NMDA RECEPTORS	TUBB3	TUBA1A, TUBB2B	MIRO1
POST-CHAPERONIN TUBULIN FOLDING PATHWAY	TUBB3	TUBB2B, TUBA1A	MIRO1
GAP JUNCTION ASSEMBLY	TUBB3	TUBB2B, TUBA1A	MIRO1
KINESINS	TUBB3	TUBB2B, TUBA1A	MIRO1

Supplementary Table 10. Idiopathic PD patient and healthy control iPCS lines used to generate midbrain organoids for previously unpublished single-cell RNA sequencing and imaging datasets. WTSI – Wellcome Sanger Institute.

Sample ID	Unique ID	Healthy/IPD	Age of onset	Age of sampling	Sex	Source
CTRL1	2.0.0.70.2.0	Healthy	-	65	F	LuxPARK cohort
CTRL2	2.0.0.72.1.0	Healthy	-	63	F	LuxPARK cohort
CTRL3	2.0.0.71.1.0	Healthy	-	68	F	LuxPARK cohort
CTRL4	2.0.0.163.0.0	Healthy	-	30	M	Coriell (GM25256)
CTRL5	2.0.0.27.0.0	Healthy	-	55	M	Coriell (GM23338)
CTRL6	2.0.0.19.0.0	Healthy	-	55-59	M	WTSI
IPD1	2.1.0.67.3.0	IPD	57	67	F	LuxPARK cohort
IPD2	2.1.0.68.2.0	IPD	62	67	F	LuxPARK cohort
IPD3	2.1.0.69.5.0	IPD	60	68	F	LuxPARK cohort
IPD4	2.1.0.64.2.0	IPD	64	68	M	LuxPARK cohort
IPD5	2.1.0.66.2.0	IPD	68	70	M	LuxPARK cohort
IPD6	2.1.0.65.9.0	IPD	59	60	M	LuxPARK cohort

Supplementary Table 11. Details on the relationship between the 18 genes involved in the ROBO signalling-associated pathways and the experimental studies.

Experimental study	Gene set	Gene number
**GBA**	PRKCA	1
**IPD**	RPS4X, RPL6, RPS7, RPL18A, LHX3, LHX4	6
**LRRK2**	ROBO2, RPS27A, RPL13, RPL38, RPL37A, RPS24, RPL27A	7
**MIRO1**	ROBO2, ROBO1, LHX2	3
**SNCA**	CXCR4, PSMD5	2

Supplementary Table 12. Drugs targeting genes involved in ROBO pathways.

Drug	Targeted proteins	Experiments
**4-PHENYLBUTYRIC ACID**	PRKCA	GBA
**ATALUREN**	RPS4X, RPL6, RPS27A, RPL13, RPL38, RPS7, RPL37A, RPL18A, RPS24, RPL27A	IPD, LRRK2
**BEVACIZUMAB-AWWB**	CXCR4, PRKCA	SNCA, GBA
**BORTEZOMIB**	PSMD5	SNCA
**CARFILZOMIB**	PSMD5	SNCA
**CISPLATIN**	CXCR4	SNCA
**DOCETAXEL ANHYDROUS**	RPL13	LRRK2
**HYDROCHLOROTHIAZIDE**	PRKCA	GBA
**INGENOL MEBUTATE**	PRKCA	GBA
**MIDOSTAURIN**	PRKCA	GBA
**PLERIXAFOR**	CXCR4	SNCA
**QUERCETIN**	PRKCA	GBA
**RESVERATROL**	PRKCA	GBA
**THALIDOMIDE**	RPL13	LRRK2
